# A highly differentiated region of wheat chromosome 7AL encodes a *Pm1a* immune receptor that recognizes its corresponding *AvrPm1a* effector from *Blumeria graminis*

**DOI:** 10.1111/nph.17075

**Published:** 2020-12-15

**Authors:** Tim Hewitt, Marion C. Müller, István Molnár, Martin Mascher, Kateřina Holušová, Hana Šimková, Lukas Kunz, Jianping Zhang, Jianbo Li, Dhara Bhatt, Raghvendra Sharma, Seraina Schudel, Guotai Yu, Burkhard Steuernagel, Sambasivam Periyannan, Brande Wulff, Mick Ayliffe, Robert McIntosh, Beat Keller, Evans Lagudah, Peng Zhang

**Affiliations:** ^1^ Agriculture & Food Commonwealth Scientific & Industrial Research Organization GPO Box 1700 Canberra ACT 2601 Australia; ^2^ School of Life and Environmental Sciences Plant Breeding Institute University of Sydney 107 Cobbitty Road Cobbitty NSW 2570 Australia; ^3^ Department of Plant and Microbial Biology University of Zurich Zollikerstrasse 107 Zürich 8008 Switzerland; ^4^ Centre of the Region Haná for Biotechnological and Agricultural Research Institute of Experimental Botany of the Czech Academy of Sciences Šlechtitelů 31 Olomouc 779 00 Czech Republic; ^5^ OT Gatersleben Leibniz Institute of Plant Genetics and Crop Plant Research Corrensstr. 3 Stadt Seeland D‐06466 Germany; ^6^ John Innes Centre Norwich, NR4 7UH UK

**Keywords:** *AvrPm* effectors, *Bgt*, *Blumeria graminis* f. sp. *tritici*, chromosome sequencing, EMS mutagenesis, NLR, *Triticum aestivum*

## Abstract

- *Pm1a*, the first powdery mildew resistance gene described in wheat, is part of a complex resistance (R) gene cluster located in a distal region of chromosome 7AL that has suppressed genetic recombination.
- A nucleotide‐binding, leucine‐rich repeat (NLR) immune receptor gene was isolated using mutagenesis and R gene enrichment sequencing (MutRenSeq). Stable transformation confirmed *Pm1a* identity which induced a strong resistance phenotype in transgenic plants upon challenge with avirulent *Blumeria graminis* (wheat powdery mildew) pathogens.
- A high‐density genetic map of a *B*.* graminis* family segregating for *Pm1a* avirulence combined with pathogen genome resequencing and RNA sequencing (RNAseq) identified *AvrPm1a* effector gene candidates. *In planta* expression identified an effector, with an N terminal Y/FxC motif, that induced a strong hypersensitive response when co‐expressed with *Pm1a* in *Nicotiana benthamiana*.
- Single chromosome enrichment sequencing (ChromSeq) and assembly of chromosome 7A suggested that suppressed recombination around the *Pm1a* region was due to a rearrangement involving chromosomes 7A, 7B and 7D. The cloning of *Pm1a* and its identification in a highly rearranged region of chromosome 7A provides insight into the role of chromosomal rearrangements in the evolution of this complex resistance cluster.

## Introduction

*Pm1*, the first catalogued wheat gene to confer resistance to powdery mildew caused by *Blumeria graminis* f. sp. *tritici* (*Bgt*), has attracted research interest for over 60 years due to its complete genetic association with both a leaf rust (caused by *Puccinia triticina*) resistance gene *Lr20* (Waterhouse, 1952) and stem rust (caused by *P*. *graminis* f. sp. *tritici*) resistance gene *Sr15* (Watson & Luig, 1966). McIntosh (1977) used ethyl methanesulfonate (EMS) mutagenesis to show that loss of *Pm1* resistance occurred independently of both *Lr20* and *Sr15* resistance, but significantly, these latter two resistances mutated together. *Pm1* was later designated *Pm1a* following the identification of four more allelic variants (*Pm1b–Pm1e*) at the *Pm1* locus (https://shigen.nig.ac.jp/wheat/komugi/genes/symbolClassList.jsp). *Pm1a*, *Pm1c* and *Pm1e* (syn. *Pm22*) were each present in common wheat *Triticum aestivum* whereas the *Pm1b* and *Pm1d* alleles were identified in *T*.* monocccum* and *T*.* spelta* var. *duhamelianum*, respectively (Hsam *et al*., 1998). Further mapping located *Pm1* in the terminal deletion bin 0.99–1.00 of chromosome 7AL, which constitutes approximately 1% of this chromosome arm (Yao *et al*., 2007; Ouyang *et al*., 2014; Worthington *et al*., 2014).

Over 60 loci for *Pm* resistance have been described in wheat and its relatives since the identification of *Pm1*. A small number of these genes have been cloned and most encode nucleotide‐binding, leucine‐rich repeat (NLR) immune receptors, i.e. *Pm2* (Sánchez‐Martín *et al*., 2016), the *Pm3* allelic series (Brunner *et al*., 2010), *Pm5e* (Xie *et al*., 2020), *Pm8* (Hurni *et al*., 2013), *Pm17* (Singh *et al*., 2018), *Pm21* (He *et al*., 2018; Xing *et al*., 2018), *Pm41* (M. Li *et al*., 2020) and *Pm60* (Zou *et al*., 2018). However, a tandem kinase‐pseudokinase, *Pm24* (Lu *et al*., 2020), also provides powdery mildew resistance as do broad‐spectrum adult plant resistance genes *Pm38* and *Pm46* that each provide partial resistance to powdery mildew and rust diseases in adult plants and encode an ABC transporter and an altered sugar transporter, respectively (Krattinger *et al*., 2009; Moore *et al*., 2015).

The corresponding avirulence effectors for several wheat NLR resistance genes, such as *Pm2* and the *Pm3* allelic series, were recently identified from *Bgt*. Map‐based cloning using haploid F_1_ populations enabled the identification of *AvrPm2*, *AvrPm3^a2/f2^*, *AvrPm3^b2/c2^* and a suppressor locus of the *Pm3* allelic series *SvrPm3^a1/f1^* (Bourras *et al*., 2015, 2019; Praz *et al*., 2017). These Avr proteins show very little sequence similarity to each other or with known proteins. However, these *Bgt* Avr proteins are small (109–130 amino acids) and may, from modelling, share some structural similarities (Praz *et al*., 2017; Bourras *et al*., 2019).

Previous attempts to isolate *Pm1* and *Lr20* by map‐based cloning were unsuccessful due to restricted genetic recombination, which may be due to a translocation from an unknown source to the distal region of chromosome 7AL (Neu *et al*., 2002). In this study, new techniques were employed to overcome this limitation for positional gene isolation. Specifically, the method of mutagenesis and resistance (R) gene enrichment sequencing (MutRenSeq) is a targeted approach that does not depend on recombination but instead utilizes a sequence bait library to enrich NLR encoding sequences from knockout mutants for the target R gene. Comparing random mutations between independent mutants allows the identification of a candidate gene. Herein, we report the successful cloning of *Pm1a* from common wheat using MutRenSeq, and the corresponding *AvrPm1a* effector from the wheat powdery mildew fungus *Bgt* using genetic mapping and RNA sequencing (RNAseq). Additionally, the method of chromosome sequencing (ChromSeq) allowed us to isolate sequences from *Pm1a* bearing chromosome 7A, showing evidence of rearrangement leading to suppressed recombination.

## Materials and Methods

### Plant materials

Six M_5_ putative point mutations for *Pm1a* in Chinese Spring*5/Axminster 7A (CS/Ax7A) substitution line carrying the *Pm1a* locus on chromosome 7A from Axminster (Sears & Briggle, 1969) were used in RenSeq analysis (mutants #396, 404, 428, 435, 446 and 650, see Supporting Information Fig. S1). F_5_ RILs used in genetic analysis and confirmation of a diagnostic marker developed from the gene candidate were derived from a combination of three different crosses segregating for *Pm1a*, namely, CS × CS/Ax7A, CS × Thew and Thew × CS. Additional mutants from Thew, along with cultivars Norka and Schomburgk were also used to test *Pm1* markers. Transformable wheat cultivar Fielder, which does not carry *Pm1*, was used for transgenic complementation. CS/Ax7A was used in flow sorting of chromosome 7A and sequencing.

### Screening for powdery mildew response

The avirulent *Bgt* inoculum used for screening *Pm1a* was collected from the glasshouse area of the CSIRO Canberra campus (Australia) and maintained on seedlings of susceptible cultivar Morocco for the duration of study. Screening of the mutagenized and genetic populations took place in an isolated glasshouse at the University of Sydney Camden Campus. Additional powdery mildew tests were similarly conducted at CSIRO Canberra with CS/Ax7A and Thew included as resistant controls. Conidiospores were shaken or brushed directly onto 2‐week‐old seedlings grown in a disease‐free glasshouse. CS/Ax7A and CS were used as resistant and susceptible controls, respectively. Phenotyping of seedlings was simply as resistant or susceptible as *Pm1a* confers a completely immune response (infection type (IT) 0) compared to CS (IT 3+).

### MutRenSeq pipeline

Treatment with EMS was performed as described in Sharp & Dong (2014) on 600 and 300 seeds of CS/Ax7A in 0.5% and 0.6% EMS, respectively. Seeds of Thew were also treated with 0.5% and 0.6% EMS using 300 seeds in each. Thus, 572 and 373 M_1_ plants of CS/Ax7A and Thew were harvested, respectively. Homozygous CS/Ax7A mutants retaining the closely linked *Lr20* and *Sr15* from six different M_2_ plants were used in subsequent R gene enrichment. DNA was prepared from leaves of uninfected seedlings of the six mutants and one wild type (WT). DNA extraction, target enrichment using Triticeae RenSeq Bait Library V2 (https://github.com/steuernb/MutantHunter), Illumina sequencing, *de novo* assembly and read mapping were carried out as described in Steuernagel *et al*. (2016). Single nucleotide polymorphism (SNP) calling and candidate identification were performed using the MuTrigo pipeline with default parameters (https://github.com/TC‐Hewitt/MuTrigo).

### *Pm1a* structure confirmation

The partial candidate contig identified by RenSeq (contig #8725) was aligned to the IWGSC Chinese Spring reference refseq v.1.0 (CSv1) (IWGSC, 2018) using Blastn (Zhang *et al*., 2000) and the top matching gene was aligned to the RenSeq WT assembly using Blastn. High scoring pairs were filtered using a custom UNIX script and the corresponding candidate contig was chosen based on presence of expected domains and a SNP in the outstanding mutant. Both candidate contigs were confirmed to belong together based on Sanger sequencing of the PCR product from genomic DNA bridging the two sequences. RNA was extracted from leaves of CS/Ax7A using the RNeasy Plant Mini Kit (Qiagen, Chadstone Centre, VIC, Australia). Transcript structure, including flanking untranslated regions (UTRs), was obtained by 5′ and 3′ RACE (rapid amplification of complementary DNA ends) using a SMARTer RACE 5′/3′ Kit (Clontech, Mountain View, CA, USA). The upstream promoter region, intron sequences and downstream terminator region were inferred from a large contig from the Ax7A *de novo* ChromSeq assembly bearing the *Pm1a* sequence. Coding sequence and translation were predicted using Fgenesh (Solovyev *et al*., 2006).

### Mutant confirmation

EMS‐induced SNPs identified in mutants by MutRenSeq were confirmed with Sanger sequencing of PCR‐amplified coding regions from mutants. Nonsynonymous amino acid substitutions were confirmed using codoncode aligner 8.0 (https://www.codoncode.com/aligner/).

### Transformation confirmation of *Pm1a*

A 9386 bp genomic sequence of *Pm1a*, including all introns, 2 kb of 5′UTR and 1.5 kb of 3′UTR encompassing the native promoter and terminator, was synthesized and cloned (Epoch Life Sciences, Missouri City, TX, USA) into *Not*I/*Sgs*I‐digested binary vector VecBarIII. The *Pm1a* gene was introduced into cv Fielder using the *Agrobacterium*‐transformation protocol (Ishida *et al*., 2015) and phosphinothricin as the selective agent. Ten independent transgenic plants (T_0_) carrying the *Pm1a* gene as well as three nontransgenic sibling control lines were recovered. The T_0_ and sibling controls were acclimatized to glasshouse conditions. After 2 weeks, sibling controls, WT controls and T_0_ plants were inoculated with *Bgt* as described earlier. Then, 10 cm leaf samples were taken from the second leaf at 10 d post‐inoculation (dpi) and again from the third or fourth leaves at 14 dpi. Leaves were scanned using an Epson Perfection V600 Photo scanner immediately after sampling. For quantification of fungal biomass, 10 and 14 dpi leaf samples were processed and stained for chitin as described by Ayliffe *et al*. (2013). Relative fluorescence was measured on a FLUOstar Omega spectrophotometer (BMG Labtech, Mornington, VIC, Australia) with excitation filter 485‐12 and emission filter Em520.

### *AvrPm1a* QTL mapping and fine mapping

Crossing, genotyping and genetic map calculations for bi‐parental population 96 224 × THUN‐12 were described in Müller *et al*. (2019). Phenotyping was performed as described in Bourras *et al*. (2019) on wheat cultivar Axminster/8*Chancellor. Susceptible cultivars Chancellor and Kanzler were used as infection controls. Virulence scoring was performed after 10 d on at least six independent detached leaf segments according to the following scoring system: 0 (0–10% leave coverage (LC)), 0.25 (10–40% LC), 0.5 (40–60% LC) and 1 (60–100% LC). Quantitative trait locus (QTL) analysis was done with the R/qtl v.1.46.2 (https://rqtl.org/) package in rstudio (RStudio‐Team, 2018). The code used to conduct the analysis as well as the input files were deposited to Github (https://github.com/MarionCMueller/QTL).

Genetic fine mapping was performed manually based on nine recombinant progeny in the QTL interval (defined by flanking markers snp64149 and snp64379) that showed virulence scores of 0 or 1. Four recombinant progeny showing intermediate phenotypes were excluded. A single recombinant progeny did not fit into our proposed high‐resolution map and would place *AvrPm1a* upstream of marker snp64348. However, this recombinant would exclude any of the candidates in the QTL interval. Given the partially quantitative nature of the *AvrPm1a* phenotype this recombinant was tentatively excluded from the analysis.

### Candidate *AvrPm1a* identification

*AvrPm1a* candidates were identified by manual curation of gene models in the candidate interval based on assembly, annotation and candidate effector definition of Bgt_genome_v3_16 reported in Müller *et al*. (2019). To verify the annotation, RNAseq data of both parental isolates were mapped against the reference genome with Star (v.2.6.0a) (Dobin *et al*., 2013) as described in Praz *et al*. (2018) and visualized with Igv (v.2.8.0) (Robinson *et al*., 2011). The RNAseq dataset was generated previously, from susceptible cv Chinese Spring and triticale cv Timbo infected with 96224 or THUN‐12, respectively (Menardo *et al*., 2016; Praz *et al*., 2018). Infected leaf samples were collected at 2 dpi during *Bgt* haustorium formation. The final set of candidate genes in the interval are listed in Supporting Information Table S1. The erroneous gene models of Bgt‐50400 and Bgt‐50399, two nonsignal peptide‐containing genes, were excluded for lacking RNAseq support or missing start codon, respectively. To identify SNPs between the parental isolates, genomic Illumina reads of isolate THUN‐12 were mapped against the reference as described in Müller *et al*. (2019). Protein domains of candidate effectors were predicted using Pfam (v.33.1) (https://pfam.xfam.org/). Signal peptide prediction was based on signalp (v.3.0) (Bendtsen *et al*., 2004) with default settings. Alignment of the E004 candidate effector family was done with the Clustal algorithm of mega x (Kumar *et al*., 2018) with default parameters. For expression analysis and differential gene expression, RNAseq reads were aligned against the *Bgt* 96224 CDS (Müller *et al*., 2019) with salmon (v.0.12.0) (Patro *et al*., 2017) as described in Praz *et al*. (2018). Subsequent expression and differential expression analysis were done using the edger (v.3.11) (Robinson *et al*., 2010) package in rstudio (RStudio‐Team, 2018) as described in Praz *et al*. (2018).

### Transient co‐expression assay in *Nicotiana benthamiana*

Effector candidate genes were codon‐optimized for expression in *Nicotiana benthamiana* using the online tool of Integrated DNA Technologies (https//eu.idtdna.com/CodonOpt). Signal peptides were predicted using the SignalP algorithm (http://www.cbs.dtu.dk/services/SignalP‐3.0/) and subsequently replaced with a start codon. For hemagglutinin (HA)‐epitope tagged effectors the corresponding sequence was added at the C‐terminus directly upstream of the stop codon. The resulting sequences predicted to encode the mature peptide, including flanking attL gateway sites, were synthesized by BioCat GmbH (https://www.biocat.com). Sequences for all synthesized DNA fragments are described in Supporting Information Dataset S1. The synthesized effector genes were subsequently cloned into the binary vector pIPKb004 (Himmelbach *et al*., 2007) using Gateway LR clonase II (Invitrogen, Carlsbad, CA, USA) according to the manufacturer’s protocol.

The *Pm1a* resistance gene was synthesized in two overlapping fragments (Dataset S1) with flanking sequences matching the binary vector pIPKb004 (Himmelbach *et al*., 2007) by BioCat GmbH. For the HA‐epitope tagged version the HA coding sequence was introduced by two subsequent rounds of PCR with the primers listed in Table S2. The two resulting fragments were cloned by In‐Fusion Cloning (Takara, Tokyo, Japan) into a modified pIPKb004 plasmid (Himmelbach *et al*., 2007) in which the gateway cassette was removed using the restriction enzymes *Hin*dIII and *Bsr*GI. All pIPKb004‐based binary constructs were verified by Sanger sequencing and transformed into *Agrobacterium tumefaciens* strain GV3101 using a freeze–thaw transformation protocol (Weigel & Glazebrook, 2006). *Agrobacterium* mediated expression in *N*.* benthamiana* and hypersensitive response (HR) assessment was performed 5 d after *Agrobacterium* infiltration according to the protocol described in Bourras *et al*. (2019).

### Protein detection

To assess whether the effector and R protein were transiently expressed in transformed *N*. *benthamiana* leaves we followed the protocol for protein extraction, sodium dodecyl sulphate–polyacrylamide gel electrophoresis (SDS‐PAGE) and Western blotting in Bourras *et al*. (2019). For HA detection we used a peroxidase‐conjugated antibody, anti‐HA‐HRP (rat monoclonal 3F10, Roche, Basel, Switzerland) at a dilution factor of 1 : 3000 in the presence of Supersignal Western blot enhancer solution (Thermo Scientific, Waltham, MA, USA) according to the manufacturer. Peroxidase‐based chemiluminescence was imaged using WesternBright ECL HRP substrate (Advansta, San Jose, CA, USA) and a Fusion FX Imaging System (Vilber Lourmat, Eberhardzell, Germany) with default settings.

### Microscopy

Infected leaf segments (3 cm) were taken at 10 and 14 dpi and treated by submersion in 1 M potassium hydroxide (KOH) and autoclaved. Treated leaves were washed and resuspended in 50 mM Tris‐HCl pH 7.5. Samples were stained with WGA‐FITC and aniline blue. Fluorescence microscopy was carried out with an AxioImager Epifluorescence Widefield Microscope (Zeiss, Oberkochen, Germany) fitted with FITC, GFP and DAPI filters.

### Fluorescence *in situ* hybridization

Fluorescence *in situ* hybridization (FISH) was carried out with oligo probes (Oligo‐pSc119.2 and Oligo‐pTa535 producing green and red signals, respectively) on CS and *Lr20*‐carrying lines (CS/Ax7A, Kenya W744, Thew, Norka) according to J. Li *et al*. (2020). Chromosome 7AL in *Lr20*‐carrying lines had a weak pSc119 signal at the distal region, whereas in CS this signal was absent. Using this karyotype, flow‐sorted chromosomes were confirmed to be 7A.

### ChromSeq and *de novo* assembly

Chromosome flow‐sorting and sequencing was performed as described in Dracatos *et al*. (2019). Briefly, 50 000 flow‐sorted chromosomes enriched for 7A (72% of the sorted fraction) were treated with proteinase K and purified on Microcon YM‐100 columns (Millipore, Bedford, MA, USA). Sequencing library, prepared from 20 ng chromosomal DNA using NEBNext^®^ Ultra™ II DNA Library Prep Kit for Illumina (New England Biolabs, Ipswich, MA, USA), was pair‐end sequenced on the Illumina NovaSeq platform, yielding 69 Gb 2 × 250 bp pair‐end reads. Ax7A sequence assemblies were generated using the contig assembly method of the Tritex pipeline (Monat *et al*., 2019). Paired‐end reads were merged with BBMerge (https://jgi.doe.gov/data‐and‐tools/bbtools/bb‐tools‐user‐guide/bbmerge‐guide/) and corrected with BFC (https://github.com/lh3/bfc). Contig assembly was executed with Minia3 (https://github.com/GATB/minia) iteratively using k‐mer sizes 100, 150, 200, 250, 300, 350, 400, 450 and 500. The *k* = 500 assembly was used for further analyses.

### Axminster 7A assembly comparison to the Chinese Spring reference

After trimming with cutadapt (https://pypi.org/project/cutadapt/), Ax7A ChromSeq reads were aligned to the CSv1 reference sequence assembly using Minimap2 (https://github.com/lh3/minimap2). Alignment records were sorted with Novosort (http://www.novocraft.com/products/novosort/) and converted to BAM format with SAMtools (Li *et al*., 2009). Read counts in nonoverlapping 1 Mb windows along the genome were determined using SAMtools and standard Unix tools. Binned read counts were imported into R and plotted along the genome using standard R functions. SNP calling was done with BCFtools (https://samtools.github.io/bcftools/). SNP positions were imported into R and binned in 5 Mb windows. SNP densities were plotted along chromosome 7A using standard R functions. The functions used in this analysis are available at https://bitbucket.org/ipk_dg_public/pm1a.

Plot of alignment scores of CSv1 chromosome 7A genes against the Ax7A assembly was created by first extracting gene sequences from CSv1 using BEDtools (https://github.com/arq5x/bedtools2) and a GFF file of CSv1 gene annotations. Genes from only chromosome 7A were aligned to the Ax7A assembly using Blastn. Outputs were filtered for top alignment over 1000 bp per query based on bit score using blast_filterv2.pyc (https://github.com/TC‐Hewitt/Misc_NGS). Bit scores were normalized to bit score/kb alignment length and alignments were ordered by physical position using a custom Unix script. Output was imported into Excel and plotted. CS IWGSC Refseq v.2.0 (CSv2) (www.wheatgenome.org) was used for coordinate‐based physical comparisons due to improved assembly accuracy over CSv1 (although annotation data were not yet available). Ax7A contig mapping to CSv2 chromosomes 2A, 3A, 7A, 7B and 7D was performed in parallel using Mash map (Jain *et al*., 2018) with identity threshold 91%, minimum segment length 5000 bp and filter mode as one‐to‐one. A custom Unix script was used to filter for best alignment per query based on the longest alignment length above identity threshold. Dotplots were generated using generated otPlot (https://github.com/marbl/MashMap). Putative breakpoints were inferred from coordinate data in filtered MashMap outputs.

### Axminster 7A assembly comparison with diploid species

Decontamination of the Ax7A assembly of likely chromosomes 2A and 3A derived contigs was achieved using previously described MashMap outputs to flag contigs having top alignments with ≥ 95% identity to either 2A or 3A. These contigs were removed from the MashMap output which was then used as an index for get_contigs.py (https://github.com/TC‐Hewitt/Misc_NGS) to extract filtered contigs to a separate Fasta file. A ‘terminal contig set’ was created by filtering the shortlisted MashMap output for contigs mapping distally to the respective breakpoints using a custom Unix script. The output was then used for contig retrieval using get_contigs.py. All ‘custom Unix scripts’ cited throughout the methods can be found at https://github.com/TC‐Hewitt/Axminster7A.

Mapping of genotyping‐by‐sequencing (GBS) data from diploid accessions was performed by first trimming raw reads using Trimmomatic (http://www.usadellab.org/cms/?page=trimmomatic). Each of the 15 species had four accessions which were concatenated into a single file per species. Mapping to the whole Ax7A assembly and ‘terminal contig set’ was performed using Bwa (Li & Durbin, 2009). The output was processed with SAMtools to remove duplicate alignments. Uniquely mapped reads were selected based on SAM tag ‘XT:A:U’ and exact matching reads were counted based on SAM tag ‘NM:i:0’. Counts were imported into Excel and plotted as a percentage of total uniquely mapped reads per species to normalize for variation in absolute reads between libraries.

### Phylogenetic tree construction

R gene protein sequences with an N‐terminal coiled‐coil domain (CNL class) were taken from the National Centre for Biotechnology Information (NCBI) database. Accession numbers are listed in Table S3. One hundred and twenty‐two sequences were aligned using muscle and a phylogenetic tree was generated using the UPGMA (unweighted pair group method with arithmetic mean) method in mega x (Kumar *et al*., 2018).

### Primer design and sequence resources

The F_5_ lines from CS × CS/Ax7A were tested with a *Pm1a*‐specific dominant sequence‐tagged site (STS) marker *Pm1aSTS1,* designed based on the positions of SNPs in the alignment of CSv1 sequences homologous to contig #8725 harbouring the *Pm1a* candidate. Primers used in this study are listed in Table S4. Genomic and transcriptomic resources used for the identification of *AvrPm1a* were reported previously: genome assembly and annotation of *Bgt* 96224 (Bgt_genome_v3_16) in Müller *et al*. (2019), re‐sequencing and RNAseq of isolate THUN‐12 in Menardo *et al*. (2016) and RNAseq of isolate 96 224 in Praz *et al*. (2018).

## Results

### Cloning of *Pm1a* by MutRenSeq

**Fig. 1:**
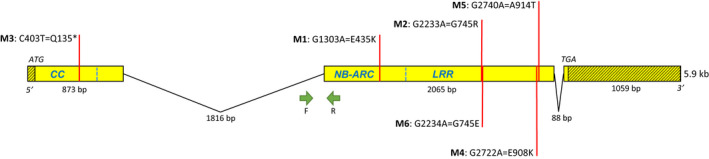
*Pm1a* gene structure showing positions of ethyl methanesulfonate (EMS) induced single nucleotide polymorphisms (SNPs) identified in mutants. SNP positions are shown by red bars labelled with mutant number (M1–M6), base change relative to coding sequence, and amino acid change. Shaded areas are UTRs. Approximate domain boundaries marked by dashed blue lines: CC, coiled‐coil; NB‐ARC, nucleotide binding‐(APAF‐1, R proteins and CED‐4); LRR, leucine rich repeat. Green arrows indicate positions of forward and reverse primers from marker *Pm1aSTS1*.

To clone *Pm1a*, we identified susceptible EMS‐generated mutants in CS/Ax7A background. Six independent mutants together with WT CS/Ax7A were processed using the MutRenSeq pipeline. Captured reads from these six lines and WT were aligned to a *de novo* reference assembly of the WT reads. One contig (#8725) of 3096 bp contained a SNP in five of the six mutants. This contig contained NB‐ARC and LRR motifs but no coiled‐coil (CC) motifs suggesting it might be a partial NLR sequence. To identify a potentially missing segment, the contig was aligned to the CSv1 reference assembly. The top hit (87.5%) was to the 3′ portion of a high confidence gene (*TraesCS7D01G540500*) predicted on chromosome 7D and functionally annotated as a disease resistance gene. The full sequence of *TraesCS7D01G540500* aligned back to the CS/Ax7A RenSeq WT assembly detected an additional 10 contigs matching the 5′ portion at 81% to 92% identity. Only one of these contigs with 91.8% identity (#3966) had a SNP in the remaining mutant and contained a CC domain. Bridging PCR was used to confirm that both contigs (#8725 and #3966) formed a single NLR gene with SNP mutations in all six mutants (Fig. S1). All SNPs were canonical for EMS mutagenesis with either C/T (one mutant) or G/A (five mutants) polymorphisms. A full transcript sequence was obtained using 5′ and 3′ RACE on RNA extracted from CS/Ax7A. SNPs were confirmed in all mutants by Sanger sequencing and determined to cause either amino acid substitutions or a premature stop (Fig. 1) in the translated protein. The full gene sequence of *Pm1a* was 5.9 kb consisting of three exons and two introns of 1816 and 88 bp. The encoded protein was 964 amino acids and contained N‐terminal CC, NB‐ARC and C‐terminal LRR domains (Fig. 1).

### The NLR gene candidate cosegregates with *Pm1a* resistance in RIL populations

A dominant STS marker, *Pm1aSTS1*, was designed from the contig #8725 sequence and amplified only in backgrounds carrying *Pm1a* (Fig. S2). The 3 kb amplicon based on the full‐length contig #8725 did not amplify in mutants that had lost both *Pm1* and *Sr15/Lr20* resistance consistent with these mutants (not used for RenSeq) containing deletions (Fig. S3). Seventy‐six CS/Ax7A F_5_ RILs and 157 CS/ Thew F_5_ RILs were then screened with the *Bgt* isolate avirulent to *Pm1a*. Resistant (*n* = 123), segregating (*n* = 6) and susceptible (*n* = 104) plants displayed clear phenotypes (Fig. S4) and the dominant *Pm1aSTS1* marker amplified only from resistant plants.

### The *Pm1a* gene candidate confers powdery mildew resistance in transformed plants

**Fig. 2:**
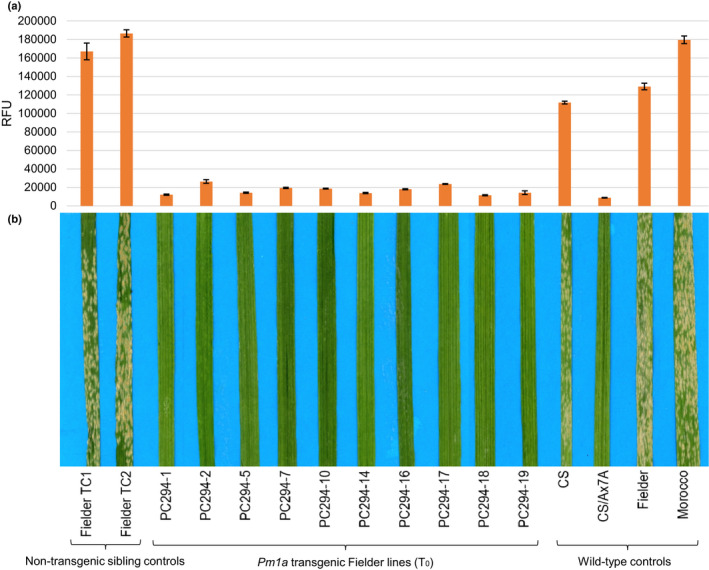
Leaves of transgenic T_0_ plants (*Triticum aestivum*) showing resistance to *Blumeria graminis* f. sp. *tritici*. (a) Fungal biomass of sampled leaves in (b) measured as relative fluorescent units (RFUs) of stained chitin with SEs of three technical replicates shown. (b) Powdery mildew responses of 10 T_0_ plants and controls at 10 d post‐inoculation (dpi). PC numbers denote independent transgenic lines. Fielder TC lines are nontransgenic tissue culture sibling controls. Resistant control: CS/Ax7A; susceptible controls: CS (Chinese Spring), Fielder, Morocco.

Susceptible cv Fielder was transformed with the *Pm1a* candidate and 10 independent transgenic lines (T_0_), with controls, were tested with *Bgt*. At both 10 and 14 dpi (not shown) all T_0_ lines showed resistance to *Bgt* similar to that observed on CS/Ax7A (Fig. 2b). The high‐level immunity was confirmed by fungal biomass measurements (Fig. 2a). The T_1_ progeny from four independent T_0_ lines were screened with *Bgt* and showed a resistant : susceptible segregation ratio of 3 : 1 or greater, indicating the presence of a stable and active *Pm1a* transgene in one or more loci of the T_0_ parents. All resistant T_1_ seedlings cosegregated with marker *Pm1aSTS1*. These data confirmed that this NLR gene conferred *Bgt* resistance in wheat and is *Pm1a*.

**Fig. 3:**
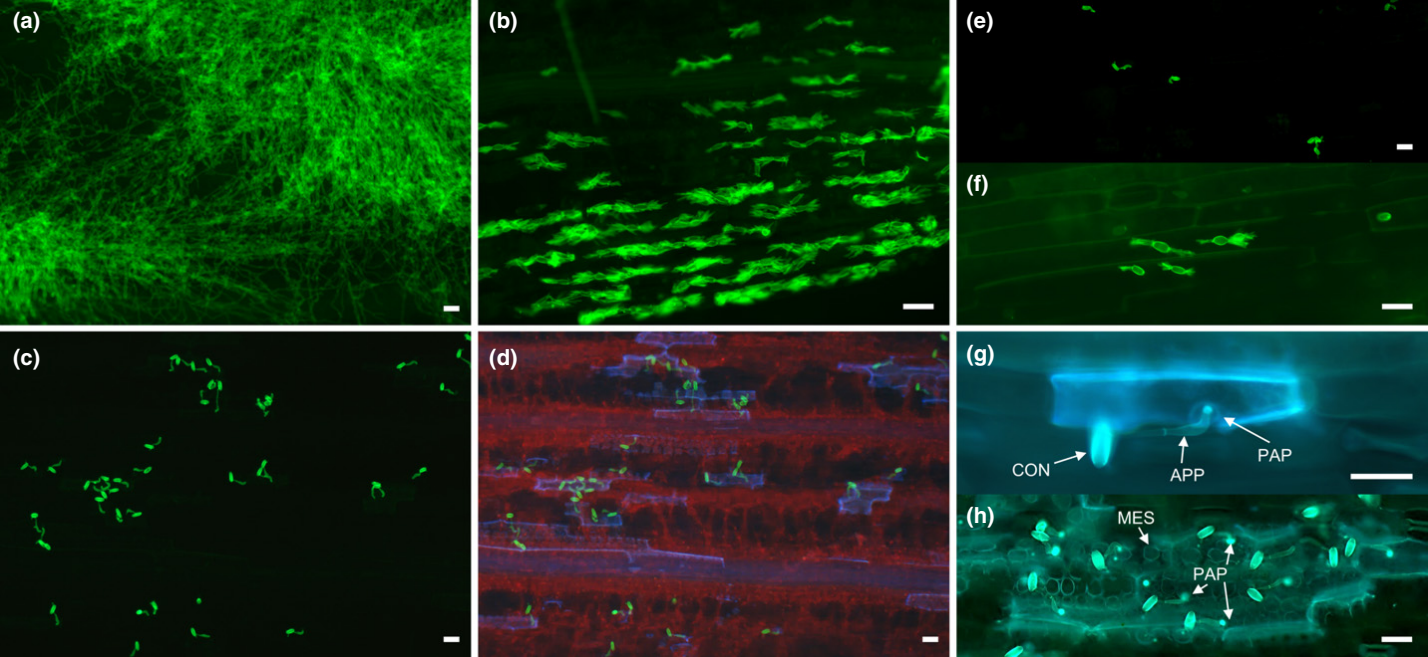
Microscopic interaction of *Blumeria graminis* f. sp. *tritici* with host (*Triticum aestivum*) on leaf surface of transgenic and nontransgenic plants at 10 d post‐inoculation. (a) Fielder showing advanced hyphal growth. (b) Secondary infection adjacent to lesion pictured in (a) showing haustoria within leaf epidermal cells. (c) Transgenic line showing germinated conidia with protruding appressoria. (d) Same image as in (c) but under UV illumination showing autofluorescence (bluish regions) at sites of infection. (e) Resistant wild‐type showing conidia similar to (c). (f) Same sample as (e) but showing some sites with haustorium development not observed in transgenics. (g) Chinese Spring/Axminster*7A epidermal cell showing autofluorescence in response to attempted penetration. Conidium (CON) projecting appressorium (APP) and papilla (PAP) forming around appressorial penetration peg. (h) Transgenic line showing a grouping of conidia inducing a cluster of autofluorescence also visible in underlying mesophylls (MES). Papillae (PAP) clearly visible as fluorescent halos indicating attempted penetration. (a–f) Stained with WGA‐FITC. (g, h) Stained with aniline blue. (a–c, e, f) Photographed with GFP filter. (d, g, h) Photographed with DAPI filter. Bars, 50 µm.

The *Pm1a* phenotype was examined microscopically in *Bgt‐*infected T_0_ leaf tissue at 10 dpi. Hyphal growth was advanced in susceptible controls (Fig. 3a) and secondary infection was evident by the presence of haustoria within epidermal cells (Fig. 3b). Conversely, on leaves of T_0_ *Pm1a* transgenics, only germinated conidiospores that produced appressoria were observed without development of conspicuous haustoria (Fig. 3c,d). Leaves from WT CS/Ax7A plants showed minimal infection similar to that of T_0_ plants (Fig. 3e), although more advanced haustorium formation was occasionally observed (Fig. 3f). Plant cell autofluorescence, indicative of host cell death (Sánchez‐Martín *et al*., 2011), was frequently associated with *Bgt* infection sites on T_0_ plants and resistant WT controls (Fig. 3d,g,h), possibly indicating HR. Autofluorescence was not associated with haustoria and secondary hyphae produced in susceptible controls.

### Cloning of *AvrPm1a*

**Fig. 4:**
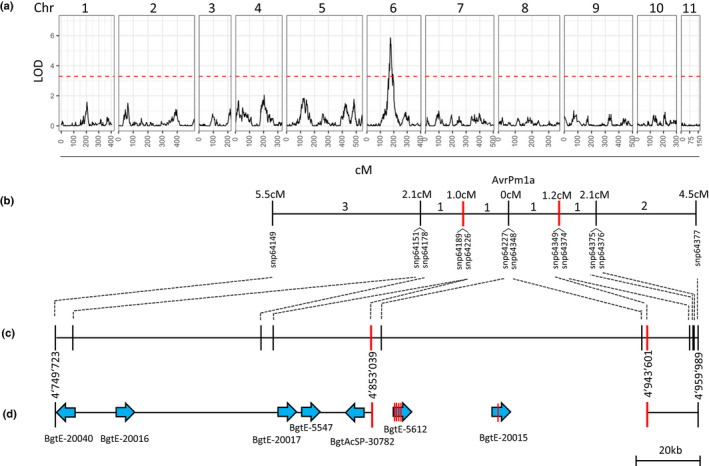
Identification of a single locus controlling avirulence on *Pm1a* in the bi‐parental mapping population *Blumeria graminis* f. sp. *tritici* (*Bgt*) 96224 (*avrPm1a*) × *B*. *g*. *triticale* THUN‐12 (*AvrPm1a*). (a) Single interval QTL mapping of the mapping population *Bgt* 96224 × *B*. *g*. *triticale* THUN‐12 on the *Pm1a* containing near isogenic line (NIL) Axminster/8*Chancellor. QTL mapping was performed on a high‐density genetic map derived from 118 F_1_ progeny based on 119 023 markers in 11 linkage groups that correspond to the 11 chromosomes of the *Bgt* isolate 96224 (Müller *et al*., 2019). The red line represents significance level of the LOD value determined by 1000 permutations. (b) Markers located in the genetic confidence interval (LOD = 1.5). Only informative markers are represented. Numbers between markers indicate the number of recombinant F_1_ individuals used for fine mapping. (c) Marker distribution on the physical interval corresponding to the genetic confidence interval determined by the QTL analysis. The final candidate interval as defined by recombinant analysis is delimited by the flanking markers depicted in red and highlighted by a yellow box. (d) Gene organization in the physical interval on chromosome 6 of the reference *Bgt* isolates 96224 in the genetic confidence interval. Candidate effector genes are depicted by blue arrows. Amino acid polymorphisms in the coding sequence of the effector genes are depicted by red lines. Details about SNPs and amino acid polymorphisms for the candidate genes can be found in Supporting Information Table S1. Gene lengths are not drawn to scale.

To identify *AvrPm1a* we used an existing mapping population with 118 sequenced haploid F_1_ progeny (Müller *et al*., 2019) originating from the cross between *B. g. triticale* isolate THUN‐12, exhibiting an avirulence phenotype on *Pm1a* near‐isogenic wheat line Axminster/8*Chancellor, and *Bgt* isolate 96224, which is virulent to *Pm1a* (Fig. S5a,b). Single interval QTL mapping with 118 progeny identified a single QTL on chromosome 6 (Logarithm of the odds (LOD) = 5.8) associated with the avirulence phenotype to *Pm1a* (Figs 4a, S5c). The genetic confidence interval (1.5 LOD) encompassed 210 266 bp in the published chromosome‐level assembly of isolate 96224 (Müller *et al*., 2019) and harboured a cluster of seven predicted effector genes (Fig. 4b–d; Table S1). Using whole‐genome re‐sequencing and RNAseq data we determined that all seven candidate effectors were present and expressed in the avirulent isolate THUN‐12 (Table S1). The high‐density genetic map and nine recombinant F_1_ progeny with clear avirulence/virulence patterns on *Pm1a* lines allowed us to further reduce the *AvrPm1a* interval to 90 562 bp and the number of candidates to two effector genes, *BgtE‐5612* and *BgtE‐20015*, located between the flanking markers (Fig. 4c,d). Both genes were strongly expressed during early infection stages of *B*. *graminis* (2 dpi) and exhibited sequence polymorphisms between the parental isolates THUN‐12 and 96224 (Fig. 4d; Table S1). Compared to the reference isolate 96224, *BgtE‐5612_THUN12* carries five SNPs resulting in four amino acid changes, whereas *BgtE‐20015_THUN12* carries a single nonsynonymous SNP (Fig. 4d), making *BgtE‐5612* and *BgtE‐20015* originating from *Pm1a* avirulent isolate THUN‐12 prime candidates for *AvrPm1a*.

**Fig. 5:**
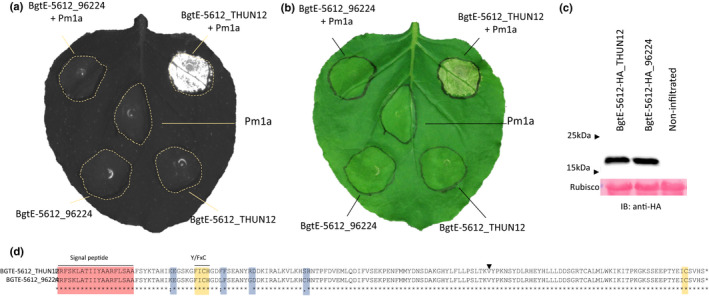
Functional validation of *AvrPm1a* in *Nicotiana benthamiana*. (a, b) Functional validation of *AvrPm1a* (*BgtE‐5612_THUN12*) in *Agrobacterium* mediated co‐expression assays in *N*. *benthamiana*. The effector candidates *BgtE‐5612_96224* and *BgtE‐5612_THUN12* and *Pm1a* were infiltrated alone (bottom) or combined (top, OD = 1.2, ratio 1 : 4 for R : effector). Hypersensitive response (HR) was assessed by fluorescence imaging ((a) Fusion FX imager, see the Materials and Methods section) or by eye (b). Photographed 5 d after infiltration. (c) Western blot detection of BGTE‐5612_THUN12 and BGTE‐5612_96224 C‐terminally fused with a hemagglutinin (HA) epitope tag (upper panel) and Ponceau staining of RuBisCO as a loading control (lower panel). HR induction of tagged BgtE‐5612 variants are shown in Supporting Information Fig. S6(d). (d) Pairwise alignment of BGTE‐5612_THUN12 and BGTE‐5612_96224 proteins. The signal peptide (red), conserved Y/FxC motif and C‐terminal cysteine (yellow), polymorphic sites (blue), and intron position (black arrow) are indicated.

Previously, *Bgt* avirulence genes were functionally validated by *Agrobacterium*‐mediated co‐expression of *NLR* genes and *Avr* candidates in the heterologous *N*. *benthamiana* system (Bourras *et al*., 2015, 2019; Praz *et al*., 2017). Following a similar approach, we optimized *BgtE‐5612* and *BgtE‐20015* codon sequences from both parental isolates for expression in *N*.* benthamiana*; synthesized genes lacking the signal peptide were tested for recognition by *Pm1a* in *Nicotiana* leaves. Co‐expression of *BgtE‐5612_THUN12* but not *BgtE‐5612_96224* with *Pm1a* in *Nicotiana* resulted in a strong HR (Figs 5a,b, S6a), whereas no such effect was observed for either version of *BgtE‐20015* (Fig. S6a). We fused all effector candidates and *Pm1a* C‐terminally with a HA epitope tag and verified protein production of all effector variants and the R protein in *Nicotiana* by Western blotting (Figs. 5c, S6b,c).

These results demonstrated that *BgtE‐5612_THUN12* is *AvrPm1a*, which is consistent with the genetic mapping data predicting the AVR component to originate from the THUN‐12 parental isolate. The recognized variant BGTE‐5612_THUN12 differs from the virulent version BGTE‐5612_96224 by four amino acids (K28E, L40F, G47R, C60S) occurring as a cluster in the N‐terminal part of the protein, surrounding the Y/FxC motif (Fig. 5d).

### Phylogenetic analysis of *Pm1a* and corresponding avirulence effector *AvrPm1a*

Comparison of the Pm1a protein sequence with a panel of 122 cloned coiled‐coil (CNL class) NLR proteins (Fig. S7) showed that Pm1a is not closely related to other known Pm proteins such as the Mla allelic series or Pm3. In fact, Pm1a is relatively dissimilar to the other NLR proteins in this panel including other wheat NLR proteins. It shows greatest similarity to Pm21 which originates from *Dasypyrum villosum* (He *et al*., 2017).

*AvrPm1a* is a member of the effector family E004, one of the 235 previously described *Bgt* and *B*. *g*. *hordei* effector families (Müller *et al*., 2019) (Fig. S13, see later). Interestingly, 22 of the 51 genes that encode E004 family proteins, including *AvrPm1a*, reside within a 900 kb genomic region on chromosome 6. This arrangement is likely to have arisen from tandem duplication and diversifying selection imposed by NLR recognition of effector sequences, such as that seen for *AvrPm1a*. In this gene family *AvrPm1a* is amongst the most highly expressed genes (Fig. S8).

AVRPM1A shows some similarity to several other cloned *Bgt* avirulence effector proteins (i.e. AVRPM2, AVRPM3^A2/F2^, AVRPM3^B2/C2^ and AVRPM3^D3^) which contain, like AVRPM1A, a signal peptide, a N‐terminal Y/FxC motif, a single, conserved cysteine towards the C‐terminus while otherwise exhibiting highly divergent amino acid sequences. Interestingly, all known *Bgt* avirulence effectors exhibit a similar exon/intron structure and are predicted to consist of a N‐terminal α‐helix followed by several β‐strands (Bourras *et al*., 2015, 2019; Praz *et al*., 2017). In the case of AVRPM1A this pattern is extended by the presence of a second α‐helix at the amino terminus, partially explaining the larger size of the effector (Figs. 5d, S14a,b, see later). Strikingly, all identified *Bgt* avirulence proteins, including AVRPM1A, exhibit an RNase‐like fold when subjected to structural modelling (Figs. 5d, S14c, see later) (Bourras *et al*., 2015 ,2019; Praz *et al*., 2017).

### Terminal chromosome 7AL is highly diverged due to rearrangement

FISH was conducted on lines CS/Ax7A, Kenya W744, Thew, and Norka carrying *Pm1‐Sr15‐Lr20*, and CS. Using Oligo‐pSc119.2 (green) and Oligo‐pTa535 (red) as probes a weak pSc119 signal was present in the distal region of chromosome 7AL in all four resistant lines but not in CS suggesting the presence of an introgression or translocation (Fig. S9).

ChromSeq was conducted on purified DNA of flow sorted chromosomes 7A of line CS/Ax7A. The chromosome sample purity was estimated to be 72% for the final DNA library with contamination primarily from chromosomes 2A and 3A. The sequencing output was *c.* 69 Gbp. A *de novo* Ax7A assembly created from the ChromSeq reads comprised of 383 563 contigs with a combined length of *c.* 1.82 Gbp and N50 of 7.85 kb.

When trimmed ChromSeq reads were aligned to the entire CSv1 reference assembly and SNP counts were plotted along chromosome 7A, few SNPs were observed in peri‐centromeric regions but a distinct increase in SNP density was observed at the distal end of 7A (Fig. S10a). This was mirrored by a discrete drop in alignment scores of CSv1 7A annotated genes aligned to the Ax7A assembly (Fig. S10b). These results indicate a clear differentiation in this distal region between chromosome 7A in the CS assembly and the sequenced Axminster 7A.

**Fig. 6:**
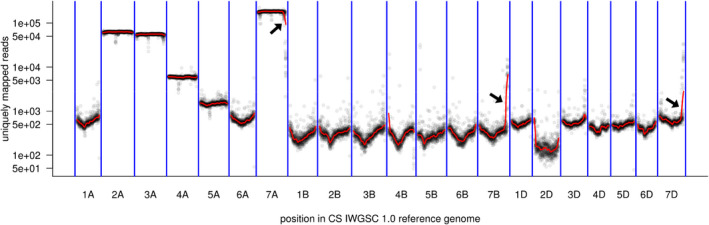
Purity of sequence reads from flow‐sorted chromosomes. Counts of uniquely mapped reads in 1 Mb bins along the *Triticum aestivum* IWGSC refseq v.1.0 reference genome. The red line is a loess smoothing. The *y*‐axis is shown in log scale. Black arrows point to steep changes in read counts at the distal ends of group 7 chromosomes.

**Fig. 7:**
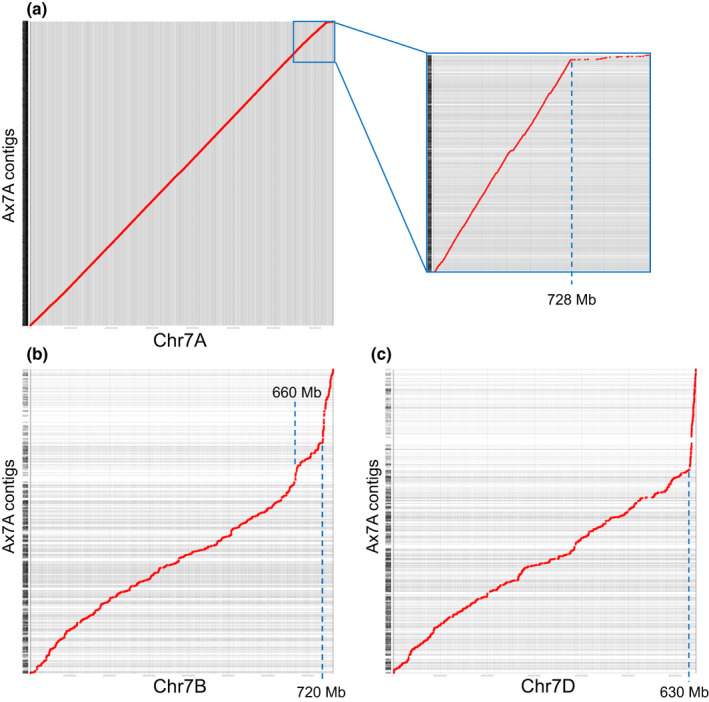
Dot plots of assembled contigs from flow‐sorted Axminster 7A chromosome mapped along group 7 chromosomes in the *Triticum aestivum* IWGSC refseq v.2.0 reference genome. Contigs (*y*‐axis) were mapped along whole chromosome assemblies (*x*‐axis). Dashed blue lines indicate approximate physical position along chromosome where there is a putative breakpoint. (a) Chromosome 7A with terminal region enlarged on the right. (b) Chromosome 7B. (c) Chromosome 7D.

Counts of uniquely mapped reads were plotted along the length of each CSv1 chromosome (Fig. 6). Majority of the reads (57%) originated from chromosome 7A, with contamination from chromosomes 2A (21%), 3A (18%) and 4A (2%). The sharp decline in read counts at the distal end of 7AL was accompanied by a concomitant rise in read counts at the distal ends of chromosomes 7BL and 7DL. When Ax7A assembly contigs were mapped to the CSv2 reference, the dot plot of 7A showed a downward inflection near the terminus indicating a decrease in homology. This placed a putative breakpoint at *c.* 728 Mb relative to CSv2 7A (Fig. 7a). Conversely, upward inflections indicating increased homology occurred at the termini of 7B and 7D, which placed putative breakpoints at *c.* 660 and 720 Mb and 630 Mb relative to CSv2 7B and 7D, respectively (Fig. 7b,c). These results suggest the terminal part of Axminster chromosome 7AL contains segments that are more related to sequences from terminal 7BL and 7DL. Based on the positions of putative breakpoints relative to CSv2 chromosomes 7A, 7B and 7D, the physical size of the linkage block on Axminster chromosome 7A may be in the range of *c.* 12.9 to 43.7 Mb.

### Possible ancestral origins of terminal 7AL

Probable contaminant contigs were removed from the Ax7A assembly based on significant alignments to either chromosome 2A or 3A in CSv2 (Fig. S11). To investigate whether the *Pm1‐Sr15‐Lr20* linkage block has potential origins in diploid wheat relatives, GBS data was sourced from an unrelated study on a collection of diploid accessions (Bernhardt *et al*., 2019). GBS reads of 15 species were aligned to the decontaminated Ax7A assembly. Unsurprisingly, *T*.* urartu*, the ancestral donor of the wheat A genome (Salamini *et al*., 2002) had the highest proportion of exact matching reads. Alignments of the other species were not noteworthy (Fig. S12a). A set of 480 contigs, provisionally representing the terminal portion of Axminster 7A, were grouped based on primary mapping after the putative breakpoints in either CSv2 7A, 7B, or 7D. To determine if any of the diploid species became more represented in the terminal portion of Axminster 7A, GBS reads similarly aligned to this terminal contig set indicated exact matching reads of *D*. *villosum* were notably higher than in the entire Ax7A alignment and were comparable to *T*. *urartu* (Fig. S12b). Given that *Pm1a* was also closest to *Pm21* in the phylogeny (Fig. S8), this raised the possibility that portions of terminal Axminster 7A were derived from *Dasypyrum* or a related lineage.

## Discussion

In this study, the *R/Avr* gene pair of *Pm1a* and *AvrPm1a* was successfully cloned using MutRenSeq and genetic mapping with RNAseq, respectively. We showed that resistance conferred by *Pm1a* can be transferred by transgenic complementation to susceptible varieties, inducing an immune reaction (Fig. 2) that limited the formation of mature haustoria in transgenic leaves. Whole cell autofluorescence in response to fungal penetration suggested a rapid onset of HR (Fig. 3). However, more rigorous staining methods are required to confirm this, and future observations of initial infection dynamics will require sampling at earlier timepoints given the rapid development of haustoria (Bushnell & Bergquist, 1974; Kunoh *et al*., 1982).

Additional *Pm* genes have been mapped to the same terminal bin as *Pm1* from various wheat relatives, notwithstanding its five purported alleles (Hsam *et al*., 1998; Singrun *et al*., 2003). These genes include *MlAG12*, *Mlm2033*, *Mlm80*, *PmTb7A*.*2*, *HSM1*, *MlUM15*, *MlWE18*, *MlIW72*, *MlIW172*, *PmU*, *Pm59*, *Pm9*, *mlRD30* and *PmG16* (Tan *et al*., 2018). Primary mapping studies placed the first 11 genes in the same marker interval, whereas the last three were placed distally (Ouyang *et al*., 2014). It was suggested that all the genes sharing the marker interval with *Pm1* were allelic to *Pm1*. However, Liang *et al*. (2016) showed that *Mlm2033* and *Mlm80* were in fact separate loci despite occupying the same interval. Most recently, the cloned gene *Pm60* (Zou *et al*., 2018) was concluded to occupy the same locus as *PmU* (Zhang *et al*., 2018), within the same interval as *Pm1*. However, sequence comparison of *Pm1a* with *Pm60* showed they were quite dissimilar (Fig. S7), so are unlikely to be homologous. This raises the question of whether the five *Pm1* alleles (*Pm1a–Pm1e*) are in fact allelic or distinct. Since the naming of these alleles was based on genetic maps covering a region of suppressed recombination, the likelihood of allelism must be questioned. Now that the *Pm1a* sequence is known, it would be highly informative to use it to probe various accessions possessing genes within this cluster to determine which are true alleles. Moreover, the cloning of additional *Pm1* alleles will allow their specificity towards *AvrPm1* to be determined.

In recent years numerous *Avr* genes have been identified in *B*. *g*. *hordei* (*Bgh*) and *Bgt* including the *Bgh*‐derived *Avra1*, *Avra7*, *Avra9*, *Avra10*, *Avra13* and *Avra22* recognized by specific alleles of the barley *Mla* resistance locus (Lu *et al*., 2016; Saur *et al*., 2019), and the *Bgt*‐derived *AvrPm2*, *AvrPm3^a2/f2^*, *AvrPm3^b2/c2^* and *AvrPm3^d3^* recognized by wheat gene *Pm2* and several *Pm3* alleles, respectively (Bourras *et al*., 2015, 2019; Praz *et al*., 2017). Interestingly, all *Blumeria* Avr proteins belong to the *Blumeria* protein superfamily of candidate secreted effector proteins (CSEPs) and exhibit common features such as a generally small size (102–130 amino acids), a signal peptide as well as a N‐terminal Y/FxC motif and a C‐terminal cysteine at a conserved location. Furthermore, numerous CSEP families, including *Avr* effectors, have a conserved exon/intron structure, indicating a common phylogenetic origin and were predicted to exhibit a RNase‐like structure (Pedersen *et al*., 2012; Bourras *et al*., 2016, 2019; Lu *et al*., 2016; Praz *et al*., 2017; Spanu, 2017 Saur *et al*., 2019). For *Bgh* CSEP BEC1054, a member of the effector family E014, which harbours both *Avra13* and *AvrPm2*, the RNase‐like structure was recently confirmed experimentally (Pennington *et al*., 2019). The newly identified *AvrPm1a* shares many of the earlier‐mentioned features of AVR proteins such as conserved sequence motifs (signal peptide, Y/FxC), a similar exon/intron structure and a predicted RNase‐fold (Figs S13, S14a,c). However, with 155 amino acids, it is significantly longer than all previously identified *Blumeria* AVRs. This difference can be attributed to the presence of an additional α‐helix at the N‐terminus and a slightly extended β‐sheet region towards the C‐terminus of the protein (Fig. S14a,b). As to how the larger effector size might influence the mode‐of‐recognition by its corresponding NLR resistance gene remains to be determined. All amino‐acid polymorphisms between the avirulent AvrPm1a_THUN12 and the virulent avrPm1a_96224 variants were located in a short stretch surrounding the Y/FxC motif. In AvrPm3^a2/f2^ effector family E008, multiple amino acid residues close to the Y/FxC motif were shown to be under diversifying selection, which was attributed to a potential selection pressure imposed by the *Pm3a* and *Pm3f* alleles (McNally *et al*., 2018). Similarly, naturally occurring sequence polymorphisms in gain of virulence alleles of *AvrPm3b2/c2* were located around the Y/FxC motif and the underlying protein region was shown to be crucial for recognition of *AvrPm3^b2/c2^* by its corresponding NLRs *Pm3b* and *Pm3c* (Bourras *et al*., 2019). The extent to which similar principles apply for recognition of *AvrPm1a* by *Pm1a* will be subject to future experiments. Cloning of additional *Pm1* alleles could lead to the identification of additional *AvrPm1* alleles, which may help reveal polymorphisms conferring gain or loss of virulence.

The sequencing of Axminster chromosome 7A is a step closer to understanding the true physical size and makeup of the terminal region of this chromosome in lines with *Pm1a* and *Sr15/Lr20*. Lines carrying the *Pm1‐Sr15‐Lr20* linkage block characteristically prohibit recombination in the terminal 7AL region. Neu *et al*. (2002) suggested this region was an alien segment or rearranged fragment. Marker assays also showed no natural recombinants in the region of interest and Australian wheat breeders were unable to combine resistance to root lesion nematode (*Rlnn1*) with a preferred white flour colour allele due to linkage with the *Psy‐A1* locus also linked with *Pm1‐Sr15‐Lr20* in certain cultivars (Jayatilake *et al*., 2013). Earlier evidence that this linkage block was due to a chromosomal rearrangement within the 7AL region was inferred from the presence of sequences that appeared to originate from the B genome (Crawford *et al*., 2011; Jayatilake, 2014). In this study, we clearly show this region to be abundant in not only sequences from 7B but also 7D (Figs 6, 7). However, precise physical ordering and composition of 7A, 7B and 7D sequences was not attainable due to significant divergence from current reference assemblies and notable disruption of synteny encompassing this region (Liang *et al*., 2016). A higher contiguity is required to achieve accurate physical resolution of Axminster 7AL, either from SMRT or Dovetail sequencing (Moll *et al*., 2017).

Comparison of terminal sequences of Axminster 7A with sequence data from diploid accessions pointed to a relatively high proportion of exact matching *D*. *villosum* reads (Fig. S12). Thus, introgression from *Dasypyrum* or a related lineage was considered. However, due to the limited number of reads available (ranging 10–60 K uniquely mapping to the terminal set), this could not be confirmed without deeper sequencing analyses or direct comparison with a genome assembly of *D*. *villosum*. The high divergence of this terminal region, which appears to be common to genotypes carrying *Pm1a* and *Lr20‐Sr15*, traces back to unrelated land varieties introduced to Australia and North America in the late 19^th^ century with no clear links between them. Ouyang *et al*. (2014) did not observe characteristic recombination suppression around *MlIW172* in *T*. *turgidum* subsp. *dicoccoides* (tetraploid). Intriguingly, Liang *et al*. (2016) did observe strong recombination suppression in this region of two *T*.* monococcum* accessions (diploid). It could be possible that an ancient introgression(s) predisposed this region to its present structure, which in turn promoted a diversification of NLRs that culminated in the linked resistance cluster carrying *Pm1a* and *Sr15‐Lr20*.

## Author contributions

TH, JZ, RM, PZ generation of mutants, disease phenotyping, genotyping, contig assembly, and data analysis; TH, BW, SP, BS, GY, EL MutRenSeq approach; IM, MM, HŠ, KH chromosome flow sorting, sequencing and *de novo* assembly; MCM, LK, SS *AvrPm1a* mapping, functional validation and bioinformatic analysis; JL cytological characterization of cultivars/lines; DB, TH, RS, MA, EL analysis of transgenics; EL, RM, BK, PZ designed and supervised the study; TH, MCM, LK, RM, EL, BK, PZ drafted the manuscript and all co‐authors provided edits. TH and MCM contributed equally to this work.

## Supporting information


**Dataset S1** Sequences produced by gene‐synthesis used in this study.

**Fig. S1** Graphs of R gene enrichment sequencing (RenSeq) mutant read alignments to candidate contigs showing mutation positions.
**Fig. S2** Marker *Pm1aSTS1* assay showing specificity to *Pm1a*‐containing *Triticum aestivum* lines.
**Fig. S3** Amplification of a large product based on the candidate contig from *Triticum aestivum* cv Chinese Spring/Axminster*7A (CS/Ax7A) was absent in double mutants of *pm1* and *sr15/lr20*.
**Fig. S4** Clear differentiation of resistant and susceptible *Triticum aestivum* seedlings in recombinant inbred line populations inoculated with *Blumeria graminis* f. sp. *tritici* (*Bgt*).
**Fig. S5** Bi‐parental mapping population *Blumeria graminis* f. sp. *tritici* (*Bgt*) 96224 (*avrPm1a*) × *B*. *g*. *triticale* THUN‐12 (*AvrPm1a*) segregates on the *Triticum aestivum* near isogenic line (NIL) Axminster/8*Chancellor containing *Pm1a*.
**Fig. S6** *Agrobacterium* mediated transient co‐expression of *BgtE‐5612* or *BgtE‐20015* with *Pm1a* and verification of protein presence in *Nicotiana benthamiana*.
**Fig. S7** Phylogenetic tree of Pm1a with cloned CNL immune receptors from various plant species.
**Fig. S8** Expression of the candidate effector family E004 gene members in the two *Blumeria graminis* isolates *B*. *g*. *triticale* THUN‐12 and *B*. *g*. *tritici* 96224 based on RNAseq data.
**Fig. S9** Cytological examination of *Triticum aestivum* chromosome 7A in cv Chinese Spring (CS) and lines carrying *Pm1a*.
**Fig. S10** *Triticum aestivum* cvs Chinese Spring (CS) vs Axminster sequence divergence in distal chromosome 7A.
**Fig. S11** Contig length distributions from assembly of *Triticum aestivum* cv Axminster flow‐sorted chromosome 7A with and without contaminants.
**Fig. S12** Proportions of reads from diploid *Triticeae* species exactly matching to the flow‐sorted chromosome 7A assembly of *Triticum aestivum* cv Axminster.
**Fig. S13** Protein alignment of the candidate effector family E004 members in the reference *Blumeria graminis* f. sp. *tritici* isolate 96224.
**Fig. S14** Alignment of *Blumeria graminis* f. sp. *tritici* AVR proteins and three family members of AVRPM1A.
**Table S1** Summary of candidate effector genes and the encoded proteins in the genetic confidence interval underlying the QTL on Bgt_chr‐06 in the reference *Blumeria graminis* f. sp. *tritici* isolate 96224.
**Table S2** Primers used to clone gene‐synthesized *Pm1a* and *Pm1a*‐HA into *Agrobacterium* compatible expression vector pIPKb004.
**Table S3** Accession numbers of immune receptor proteins used to construct the phylogenetic tree in Fig. S7.
**Table S4** Primers used for PCR and sequencing of *Pm1a*.
Please note: Wiley Blackwell are not responsible for the content or functionality of any Supporting Information supplied by the authors. Any queries (other than missing material) should be directed to the *New Phytologist* Central Office.

## Data Availability

All high‐throughput RenSeq and ChromSeq sequencing data described in this article have been deposited under BioProject PRJEB39498. *Pm1a* sequence is available under GenBank accession MW531535. [Correction added after online publication 15 December 2020: an accession number was added in the preceding sentence.] *AvrPm1a* variant 96224 and variant THUN‐12 sequences are available under GenBank accession nos. MT773601 and MT773602, respectively. GBS sequencing data for diploid accessions came from the study by Bernhardt *et al*. (2019) and can be accessed at http://dx.doi.org/10.5447/IPK/2019/18.

## References

[nph17075-bib-0001] Ayliffe M , Periyannan SK , Feechan A , Dry I , Schumann U , Wang MB , Pryor A , Lagudah E . 2013. A simple method for comparing fungal biomass in infected plant tissues. Molecular Plant–Microbe Interactions 26: 658–667. 23405866 10.1094/MPMI-12-12-0291-R

[nph17075-bib-0002] Bendtsen JD , Nielsen H , von Heijne G , Brunak S . 2004. Improved prediction of signal peptides: SignalP 3.0. Journal of Molecular Biology 340: 783–795. 15223320 10.1016/j.jmb.2004.05.028

[nph17075-bib-0003] Bernhardt N , Brassac J , Dong X , Willing EM , Poskar CH , Kilian B , Blattner FR . 2019. Genome‐wide sequence information reveals recurrent hybridization among diploid wheat wild relatives. The Plant Journal 102: 493–506. 10.1111/tpj.14641 31821649

[nph17075-bib-0004] Bourras S , Kunz L , Xue MF , Praz CR , Muller MC , Kalin C , Schlafli M , Ackermann P , Fluckiger S , Parlange F *et al*. 2019. The *AvrPm3‐Pm3* effector‐NLR interactions control both race‐specific resistance and host‐specificity of cereal mildews on wheat. Nature Communications 10: 2292. 10.1038/s41467-019-10274-1 PMC6533294 31123263

[nph17075-bib-0005] Bourras S , McNally KE , Ben‐David R , Parlange F , Roffler S , Praz CR , Oberhaensli S , Menardo F , Stirnweis D , Frenkel Z *et al*. 2015. Multiple avirulence loci and allele‐specific effector recognition control the *Pm3* race‐specific resistance of wheat to powdery mildew. The Plant Cell 27: 2991–3012. 26452600 10.1105/tpc.15.00171 PMC4682313

[nph17075-bib-0006] Bourras S , McNally KE , Mueller MC , Wicker T , Keller B . 2016. Avirulence genes in cereal powdery mildews: the gene‐for‐gene hypothesis 2.0. Frontiers in Plant Science 7: 241. 26973683 10.3389/fpls.2016.00241 PMC4771761

[nph17075-bib-0007] Brunner S , Hurni S , Streckeisen P , Mayr G , Albrecht M , Yahiaoui N , Keller B . 2010. Intragenic allele pyramiding combines different specificities of wheat *Pm3* resistance alleles. The Plant Journal 64: 433–445. 20804457 10.1111/j.1365-313X.2010.04342.x

[nph17075-bib-0008] Bushnell WR , Bergquist SE . 1974. Aggregation of host cytoplasm and the formation of papillae and haustoria in powdery mildew of barley. Phytopathology 65: 310–318.

[nph17075-bib-0009] Crawford AC , Stefanova K , Lambe W , McLean R , Wilson R , Barclay I , Francki MG . 2011. Functional relationships of phytoene synthase 1 alleles on chromosome 7A controlling flour colour variation in selected Australian wheat genotypes. Theoretical and Applied Genetics 123: 95–108. 21442411 10.1007/s00122-011-1569-9

[nph17075-bib-0010] Dobin A , Davis CA , Schlesinger F , Drenkow J , Zaleski C , Jha S , Batut P , Chaisson M , Gingeras TR . 2013. STAR: ultrafast universal RNA‐seq aligner. Bioinformatics 29: 15–21. 23104886 10.1093/bioinformatics/bts635 PMC3530905

[nph17075-bib-0011] Dracatos PM , Bartoš J , Elmansour H , Singh D , Karafiátová M , Zhang P , Steuernagel B , Svačina R , Cobbin JCA , Clark B *et al*. 2019. The Coiled‐Coil NLR *Rph1*, confers leaf rust resistance in barley cultivar Sudan. Plant Physiology 179: 1362–1372. 30593453 10.1104/pp.18.01052 PMC6446784

[nph17075-bib-0012] He H , Ji Y , Zhu S , Li B , Zhao R , Jiang Z , Bie T . 2017. Genetic, physical and comparative mapping of the powdery mildew resistance gene *Pm21* originating from *Dasypyrum villosum* . Frontiers in Plant Science 8: 1914. 29163626 10.3389/fpls.2017.01914 PMC5681962

[nph17075-bib-0013] He H , Zhu S , Zhao R , Jiang Z , Ji Y , Ji J , Qiu D , Li H , Bie T . 2018. *Pm21*, encoding a typical CC‐NBS‐LRR protein, confers broad‐spectrum resistance to wheat powdery mildew disease. Molecular Plant 11: 879–882. 29567454 10.1016/j.molp.2018.03.004

[nph17075-bib-0014] Himmelbach A , Zierold U , Hensel G *et al*. 2007. A set of modular binary vectors for transformation of cereals. Plant Physiology 145: 1192–1200. 17981986 10.1104/pp.107.111575 PMC2151723

[nph17075-bib-0015] Hsam KSL , Huang QX , Ernst F , Hartl L , Zeller JF . 1998. Chromosomal location of genes for resistance to powdery mildew in common wheat (*Triticum aestivum* L. em Thell.). 5. Alleles at the *Pm1* locus. Theoretical and Applied Genetics 96: 1129–1134.

[nph17075-bib-0016] Hurni S , Brunner S , Buchmann G , Herren G , Jordan T , Krukowski P , Wicker T , Yahiaoui N , Mago R , Keller B . 2013. Rye *Pm8* and wheat *Pm3* are orthologous genes and show evolutionary conservation of resistance function against powdery mildew. The Plant Journal 76: 957–969. 24124925 10.1111/tpj.12345

[nph17075-bib-0017] International Wheat Genome Sequencing Consortium (IWGSC) . 2018. Shifting the limits in wheat research and breeding using a fully annotated reference genome. Science 361: eaar7191. 30115783 10.1126/science.aar7191

[nph17075-bib-0018] Ishida Y , Tsunashima M , Hiei Y , Komari T . 2015. Wheat (*Triticum aestivum* L.) transformation using immature embryos. In Wang K ed. Agrobacterium protocols: vol. 1. New York, NY, USA: Springer, 189–198. 10.1007/978-1-4939-1695-5_15 25300841

[nph17075-bib-0019] Jain C , Koren S , Dilthey A , Phillippy AM , Aluru S . 2018. A fast adaptive algorithm for computing whole‐genome homology maps. Bioinformatics 34: 748–756. 30423094 10.1093/bioinformatics/bty597 PMC6129286

[nph17075-bib-0020] Jayatilake DV . 2014. *Fine mapping of nematode resistance genes* Rlnn1 *and* Cre8 *in wheat (*Triticum aestivum*)* . PhD thesis, University of Adelaide, Adelaide, SA, Australia.

[nph17075-bib-0021] Jayatilake DV , Tucker EJ , Bariana H , Kuchel H , Edwards J , McKay AC , Chalmers K , Mather DE . 2013. Genetic mapping and marker development for resistance of wheat against the root lesion nematode *Pratylenchus neglectus* . BMC Plant Biology 13: 230. 24377498 10.1186/1471-2229-13-230 PMC3923441

[nph17075-bib-0022] Krattinger SG , Lagudah ES , Spielmeyer W , Singh RP , Huerta‐Espino J , McFadden H , Bossolini E , Selter LL , Keller B . 2009. A putative ABC transporter confers durable resistance to multiple fungal pathogens in wheat. Science 323: 1360–1363. 19229000 10.1126/science.1166453

[nph17075-bib-0023] Kumar S , Stecher G , Li M , Knyaz C , Tamura K . 2018. MEGA X: molecular evolutionary genetics analysis across computing platforms. Molecular Biology and Evolution 35: 1547–1549. 29722887 10.1093/molbev/msy096 PMC5967553

[nph17075-bib-0024] Kunoh H , Yamamori K , Ishizaki H . 1982. Cytological studies of early stages of powdery mildew in barley and wheat. VIII. Autofluorescence at penetration sites of *Erysiphe graminis hordei* on living barley coleoptiles. Physiological Plant Pathology 21: 373–379.

[nph17075-bib-0025] Li H , Durbin R . 2009. Fast and accurate short read alignment with Burrows–Wheeler transform. Bioinformatics 25: 1754–1760. 19451168 10.1093/bioinformatics/btp324 PMC2705234

[nph17075-bib-0026] Li H , Handsaker B , Wysoker A , Fennell T , Ruan J , Homer N , Marth G , Abecasis G , Durbin R . 2009. The sequence alignment/map format and SAMtools. Bioinformatics 25: 2078–2079. 19505943 10.1093/bioinformatics/btp352 PMC2723002

[nph17075-bib-0027] Li J , Dundas I , Dong C , Li G , Trethowan R , Yang Z , Hoxha S , Zhang P . 2020. Identification and characterization of a new stripe rust resistance gene *Yr83* on rye chromosome 6R in wheat. Theoretical and Applied Genetics 133: 1095–1107. 31955232 10.1007/s00122-020-03534-y

[nph17075-bib-0028] Li M , Dong L , Li B , Wang Z , Xie J , Qiu D , Li Y , Shi W , Yang L , Wu Q *et al*. 2020. A CNL protein in wild emmer wheat confers powdery mildew resistance. New Phytologist 228: 1027–1037. 10.1111/nph.16761 32583535

[nph17075-bib-0029] Liang JC , Fu BS , Tang WB , Khan NU , Li N , Ma ZQ . 2016. Fine mapping of two wheat powdery mildew resistance genes located at the *Pm1* cluster. Plant Genome 9: 1–9. 10.3835/plantgenome2015.09.0084 27898804

[nph17075-bib-0030] Lu P , Guo L , Wang Z , Li B , Li J , Li Y , Qiu D , Shi W , Yang L , Wang N *et al*. 2020. A rare gain of function mutation in a wheat tandem kinase confers resistance to powdery mildew. Nature Communications 11: 680. 10.1038/s41467-020-14294-0 PMC6997164 32015344

[nph17075-bib-0031] Lu XL , Kracher B , Saur IML , Bauer S , Ellwood SR , Wise R , Yaeno T , Maekawa T , Schulze‐Lefert P . 2016. Allelic barley MLA immune receptors recognize sequence‐unrelated avirulence effectors of the powdery mildew pathogen. Proceedings of the National Academy of Sciences USA 113: 6486–6495. 10.1073/pnas.1612947113 PMC5081590 27702901

[nph17075-bib-0032] McIntosh RA . 1977. Nature of induced mutations affecting disease reaction in wheat. Induced mutations against plant disease. Vienna, Austria: International Atomic Energy Agency. 551–564.

[nph17075-bib-0033] McNally KE , Menardo F , Luthi L , Praz CR , Muller MC , Kunz L , Ben‐David R , Chandrasekhar K , Dinoor A , Cowger C *et al*. 2018. Distinct domains of the *AvrPm3* ^(A2/F2)^ avirulence protein from wheat powdery mildew are involved in immune receptor recognition and putative effector function. New Phytologist 218: 681–695. 10.1111/nph.15026 PMC6175116 29453934

[nph17075-bib-0034] Menardo F , Praz CR , Wyder S , Ben‐David R , Bourras S , Matsumae H , McNally KE , Parlange F , Riba A , Roffler S *et al*. 2016. Hybridization of powdery mildew strains gives rise to pathogens on novel agricultural crop species. Nature Genetics 48: 201–205. 26752267 10.1038/ng.3485

[nph17075-bib-0035] Moll KM , Zhou P , Ramaraj T , Fajardo D , Devitt NP , Sadowsky MJ , Stupar RM , Tiffin P , Miller JR , Young ND *et al*. 2017. Strategies for optimizing BioNano and Dovetail explored through a second reference quality assembly for the legume model *Medicago truncatula* . BMC Genomics 18: 578. 28778149 10.1186/s12864-017-3971-4 PMC5545040

[nph17075-bib-0036] Monat C , Padmarasu S , Lux T , Wicker T , Gundlach H , Himmelbach A , Ens J , Li C , Muehlbauer GJ , Schulman AH *et al*. 2019. TRITEX: chromosome‐scale sequence assembly of *Triticeae* genomes with open‐source tools. Genome Biology 20: 284. 31849336 10.1186/s13059-019-1899-5 PMC6918601

[nph17075-bib-0037] Moore JW , Herrera‐Foessel S , Lan CX , Schnippenkoetter W , Ayliffe M , Huerta‐Espino J , Lillemo M , Viccars L , Milne R , Periyannan S *et al*. 2015. A recently evolved hexose transporter variant confers resistance to multiple pathogens in wheat. Nature Genetics 47: 1494. 26551671 10.1038/ng.3439

[nph17075-bib-0038] Müller MC , Praz CR , Sotiropoulos AG , Menardo F , Kunz L , Schudel S , Oberhänsli S , Poretti M , Wehrli A , Bourras S *et al*. 2019. A chromosome‐scale genome assembly reveals a highly dynamic effector repertoire of wheat powdery mildew. New Phytologist 221: 2176–2189. 10.1111/nph.15529 PMC6587952 30388298

[nph17075-bib-0039] Neu C , Stein N , Keller B . 2002. Genetic mapping of the *Lr20‐Pm1* resistance locus reveals suppressed recombination on chromosome arm 7AL in hexaploid wheat. Genome 45: 737–744. 12175077 10.1139/g02-040

[nph17075-bib-0041] Ouyang S , Zhang D , Han J , Zhao X , Cui Y , Song W , Huo N , Liang Y , Xie J , Wang Z *et al*. 2014. Fine physical and genetic mapping of powdery mildew resistance gene *MlIW172* originating from wild emmer (*Triticum dicoccoides*). PLoS ONE 9: e100160. 24955773 10.1371/journal.pone.0100160 PMC4067302

[nph17075-bib-0042] Patro R , Duggal G , Love MI , Irizarry RA , Kingsford C . 2017. Salmon provides fast and bias‐aware quantification of transcript expression. Nature Methods 14: 417. 28263959 10.1038/nmeth.4197 PMC5600148

[nph17075-bib-0043] Pedersen C , van Themaat EVL , McGuffin LJ , Abbott JC , Burgis TA , Barton G , Bindschedler LV , Lu XL , Maekawa T , Wessling R *et al*. 2012. Structure and evolution of barley powdery mildew effector candidates. BMC Genomics 13: 694. 23231440 10.1186/1471-2164-13-694 PMC3582587

[nph17075-bib-0044] Pennington HG , Jones R , Kwon S , Bonciani G , Thieron H , Chandler T , Luong P , Morgan SN , Przydacz M , Bozkurt T *et al*. 2019. The fungal ribonuclease‐like effector protein CSEP0064/BEC1054 represses plant immunity and interferes with degradation of host ribosomal RNA. PLoS Pathogens 15: e1007620. 30856238 10.1371/journal.ppat.1007620 PMC6464244

[nph17075-bib-0045] Praz CR , Bourras S , Zeng FS , Sanchez‐Martin J , Menardo F , Xue MF , Yang LJ , Roffler S , Boni R , Herren G *et al*. 2017. *AvrPm2* encodes an RNase‐like avirulence effector which is conserved in the two different specialised forms of wheat and rye powdery mildew fungus. New Phytologist 213: 1301–1314. 10.1111/nph.14372 PMC5347869 27935041

[nph17075-bib-0046] Praz CR , Menardo F , Robinson MD , Müller MC , Wicker T , Bourras S , Keller B . 2018. Non‐parent of origin expression of numerous effector genes indicates a role of gene regulation in host adaption of the hybrid triticale powdery mildew pathogen. Frontiers in Plant Science 9: 49. 29441081 10.3389/fpls.2018.00049 PMC5797619

[nph17075-bib-0047] Robinson JT , Thorvaldsdottir H , Winckler W , Guttman M , Lander ES , Getz G , Mesirov JP . 2011. Integrative genomics viewer. Nature Biotechnology 29: 24–26. 10.1038/nbt.1754 PMC3346182 21221095

[nph17075-bib-0048] Robinson MD , McCarthy DJ , Smyth GK . 2010. edgeR: a Bioconductor package for differential expression analysis of digital gene expression data. Bioinformatics 26: 139–140. 19910308 10.1093/bioinformatics/btp616 PMC2796818

[nph17075-bib-0049] RStudio Team . 2018. *RStudio: Integrated development environment for R* v.1.2.1335. [WWW document] URL http://www.rstudio.com/.

[nph17075-bib-0050] Salamini F , Ozkan H , Brandolini A , Schafer‐Pregl R , Martin W . 2002. Genetics and geography of wild cereal domestication in the near east. Nature Reviews Genetics 3: 429–441. 10.1038/nrg817 12042770

[nph17075-bib-0051] Sánchez‐Martín J , Rubiales D , Prats E . 2011. Resistance to powdery mildew (*Blumeria graminis* f. sp. *avenae*) in oat seedlings and adult plants. Plant Pathology 60: 846–856.

[nph17075-bib-0052] Sánchez‐Martín J , Steuernagel B , Ghosh S , Herren G , Hurni S , Adamski N , Vrána J , Kubaláková M , Krattinger SG , Wicker T *et al*. 2016. Rapid gene isolation in barley and wheat by mutant chromosome sequencing. Genome Biology 17: 221. 27795210 10.1186/s13059-016-1082-1 PMC5087116

[nph17075-bib-0053] Saur IML , Bauer S , Kracher B , Lu XL , Franzeskakis L , Muller MC , Sabelleck B , Kummel F , Panstruga R , Maekawa T *et al*. 2019. Multiple pairs of allelic MLA immune receptor‐powdery mildew AVR(A) effectors argue for a direct recognition mechanism. eLife 8: e44471. 30777147 10.7554/eLife.44471 PMC6414202

[nph17075-bib-0054] Sears ER , Briggle LW . 1969. Mapping gene *Pm1* for resistance to *Erysiphe graminis* f. sp. *tritici* on chromosome 7A of wheat. Crop Science 9: 96.

[nph17075-bib-0055] Sharp P , Dong C . 2014. TILLING for Plant Breeding. In: Fleury D , Whitford R , eds. Crop breeding methods and protocols. New York, NY, USA: Springer Protocols. 155–165. 10.1007/978-1-4939-0446-4_13 24816667

[nph17075-bib-0056] Singh SP , Hurni S , Ruinelli M , Brunner S , Sanchez‐Martin J , Krukowski P , Peditto D , Buchmann G , Zbinden H , Keller B . 2018. Evolutionary divergence of the rye *Pm17* and *Pm8* resistance genes reveals ancient diversity. Plant Molecular Biology 98: 249–260. 30244408 10.1007/s11103-018-0780-3

[nph17075-bib-0057] Singrun C , Hsam SL , Hartl L , Zeller FJ , Mohler V . 2003. Powdery mildew resistance gene *Pm22* in cultivar Virest is a member of the complex *Pm1* locus in common wheat (*Triticum aestivum* L. em Thell.). Theoretical and Applied Genetics 106: 1420–1424. 12750784 10.1007/s00122-002-1187-7

[nph17075-bib-0058] Solovyev V , Kosarev P , Seledsov I , Vorobyev D . 2006. Automatic annotation of eukaryotic genes, pseudogenes and promoters. Genome Biology 7(Suppl. 1): S10.1‐S10.12. 16925832 10.1186/gb-2006-7-s1-s10 PMC1810547

[nph17075-bib-0059] Spanu PD . 2017. Cereal immunity against powdery mildews targets RNase‐Like Proteins associated with Haustoria (RALPH) effectors evolved from a common ancestral gene. New Phytologist 213: 969–971. 10.1111/nph.14386 28079937

[nph17075-bib-0060] Steuernagel B , Periyannan SK , Hernandez‐Pinzon I , Witek K , Rouse MN , Yu G , Hatta A , Ayliffe M , Bariana H , Jones JD *et al*. 2016. Rapid cloning of disease‐resistance genes in plants using mutagenesis and sequence capture. Nature Biotechnology 34: 652–655. 10.1038/nbt.3543 27111722

[nph17075-bib-0061] Tan C , Li G , Cowger C , Carver BF , Xu X . 2018. Characterization of *Pm59*, a novel powdery mildew resistance gene in Afghanistan wheat landrace PI 181356. Theoretical and Applied Genetics 131: 1145–1152. 29453526 10.1007/s00122-018-3067-9

[nph17075-bib-0063] Waterhouse WL . 1952. Australian rust studies. IX. Physiological race determinations and surveys of cereal rusts. Proceedings of the Linnean Society of New South Wales 77: 209–258.

[nph17075-bib-0064] Watson IA , Luig NH . 1966. SR 15 – a new gene for use in the classification of *Puccinia graminis* var. *tritici* . Euphytica 15: 239–247.

[nph17075-bib-0065] Weigel D , Glazebrook J . 2006. Transformation of *Agrobacterium* using the freeze–thaw method. CSH protocols 7: pdb.prot4666. 10.1101/pdb.prot4666 22484682

[nph17075-bib-0066] Worthington M , Lyerly J , Petersen S , Brown‐Guedira G , Marshall D , Cowger C , Parks R , Murphy JP . 2014. *MlUM15*: an *Aegilops neglecta*‐derived powdery mildew resistance gene in common wheat. Crop Science 54: 1397–1406.

[nph17075-bib-0067] Xie J , Guo G , Wang Y , Hu T , Wang L , Li J , Qiu D , Li Y , Wu Q , Lu P *et al*. 2020. A rare single nucleotide variant in *Pm5e* confers powdery mildew resistance in common wheat. New Phytologist 228: 1011–1026. 10.1111/nph.16762 32569398

[nph17075-bib-0068] Xing L , Hu P , Liu J , Witek K , Zhou S , Xu J , Zhou W , Gao L , Huang Z , Zhang R *et al*. 2018. *Pm21* from *Haynaldia villosa* encodes a CC‐NBS‐LRR protein conferring powdery mildew resistance in wheat. Molecular Plant 11: 874–878. 29567451 10.1016/j.molp.2018.02.013

[nph17075-bib-0069] Yao G , Zhang J , Yang L , Xu H , Jiang Y , Xiong L , Zhang C , Zhang Z , Ma Z , Sorrells ME . 2007. Genetic mapping of two powdery mildew resistance genes in einkorn (*Triticum monococcum* L.) accessions. Theoretical and Applied Genetics 114: 351–358. 17091263 10.1007/s00122-006-0438-4

[nph17075-bib-0070] Zhang L , Zheng XW , Qiao LY , Qiao L , Zhao JJ , Wang JM , Zheng J . 2018. Analysis of three types of resistance gene analogs in *PmU* region from *Triticum urartu* . Journal of Integrative Agriculture 17: 2601–2611.

[nph17075-bib-0071] Zhang Z , Schwartz S , Wagner L , Miller W . 2000. A greedy algorithm for aligning DNA sequences. Journal of Computational Biology 7: 203–214. 10890397 10.1089/10665270050081478

[nph17075-bib-0072] Zou S , Wang H , Li Y , Kong Z , Tang D . 2018. The NB‐LRR gene *Pm60* confers powdery mildew resistance in wheat. New Phytologist 218: 298–309. 10.1111/nph.14964 29281751

