# Supplementary material for: A highly differentiated region of wheat chromosome 7AL encodes a Pm1a immune receptor that recognizes its corresponding AvrPm1a effector from Blumeria graminis
Source: New Phytol. 2020 Dec 15;229(5):2812–26. doi: 10.1111/nph.17075 (PMC8022591; doi:10.1111/nph.17075)
Supplement: Supplementary file 2 — Fig. S1 Graphs of R gene enrichment sequencing (RenSeq) mutant read alignments to candidate contigs showing mutation positions. Fig. S2 Marker Pm1aSTS1 assay showing specificity to Pm1a‐containing Triticum aestivum lines. Fig. S3 Amplification of a large product based on the candidate contig from Triticum aestivum cv Chinese Spring/Axminster*7A (CS/Ax7A) was absent in double mutants of pm1 and sr15/lr20. Fig. S4 Clear differentiation of resistant and susceptible Triticum aestivum seedlings in recombinant inbred line populations inoculated with Blumeria graminis f. sp. tritici (Bgt). Fig. S5 Bi‐parental mapping population Blumeria graminis f. sp. tritici (Bgt) 96224 (avrPm1a) × B. g. triticale THUN‐12 (AvrPm1a) segregates on the Triticum aestivum near isogenic line (NIL) Axminster/8*Chancellor containing Pm1a. Fig. S6 Agrobacterium mediated transient co‐expression of BgtE‐5612 or BgtE‐20015 with Pm1a and verification of protein presence in Nicotiana benthamiana. Fig. S7 Phylogenetic tree of Pm1a with cloned CNL immune receptors from various plant species. Fig. S8 Expression of the candidate effector family E004 gene members in the two Blumeria graminis isolates B. g. triticale THUN‐12 and B. g. tritici 96224 based on RNAseq data. Fig. S9 Cytological examination of Triticum aestivum chromosome 7A in cv Chinese Spring (CS) and lines carrying Pm1a. Fig. S10 Triticum aestivum cvs Chinese Spring (CS) vs Axminster sequence divergence in distal chromosome 7A. Fig. S11 Contig length distributions from assembly of Triticum aestivum cv Axminster flow‐sorted chromosome 7A with and without contaminants. Fig. S12 Proportions of reads from diploid Triticeae species exactly matching to the flow‐sorted chromosome 7A assembly of Triticum aestivum cv Axminster. Fig. S13 Protein alignment of the candidate effector family E004 members in the reference Blumeria graminis f. sp. tritici isolate 96224. Fig. S14 Alignment of Blumeria graminis f. sp. tritici AVR proteins and three family memb [file NPH-229-2812-s001.pdf]

**New Phytologist Supporting Information**

**Article title:** A highly differentiated region of wheat chromosome 7AL encodes a *Pm1a* immune receptor that recognises its corresponding *AvrPm1a* effector from *Blumeria graminis*

**Authors:** Tim Hewitt, Marion C. Mueller, István Molnár , Martin Mascher, Kateřina Holušová, Hana Šimková, Lukas Kunz, Jianping Zhang, Jianbo Li, Dhara Bhatt, Raghvendra Sharma, Seraina Schudel, Guotai Yu, Burkhard Steuernagel, Sambasivam Periyannan, Brande Wulff, Mick Ayliffe, Robert McIntosh, Beat Keller, Evans Lagudah and Peng Zhang

**Article acceptance date:** 1 November 2020

**Fig. S1** Graphs of R gene enrichment sequencing (RenSeq) mutant read alignments to candidate contigs showing mutation positions. Histograms showing coverage depth of read alignments to candidate contigs juxtaposed. Blue graphs are mutants with IDs labelled on the left. Grey graph is parental wild-type Chinese Spring/Axminster*7A (CS/Ax7A). SNPs are denoted with red or green vertical bars that indicate a mismatch with WT CS/Ax7A occurring in almost 100% of the overlapping reads. Specific base changes are labelled. The motifs panel indicates predicted R gene motifs and how they fit into encoded protein domains and exon structure. Domains: CC, coiled-coil; P-loop, phosphate-binding loop; NB-ARC, nucleotide binding-(APAF-1, R proteins and CED-4); LRR, leucine rich repeat.

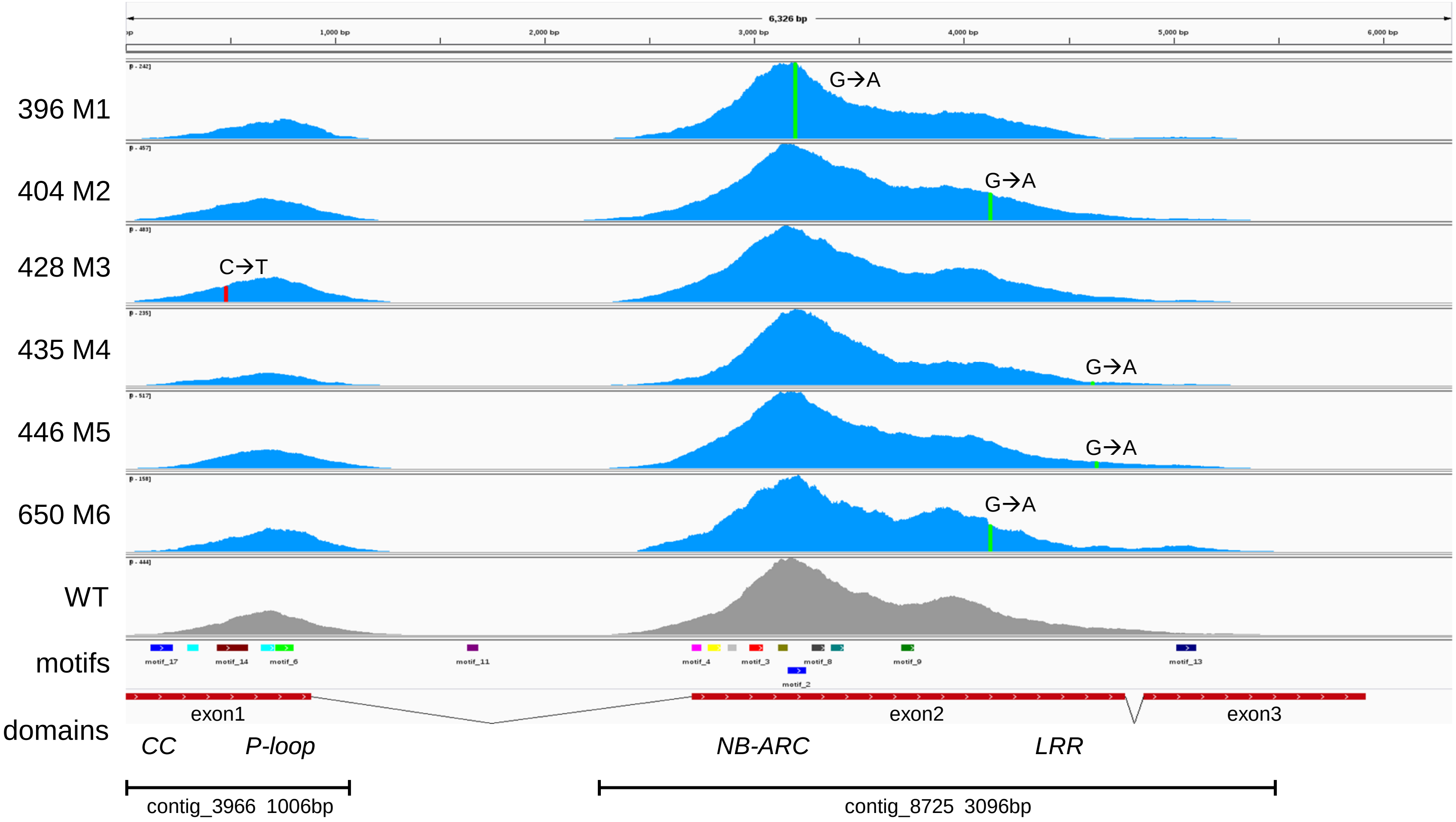

**Fig. S2** Marker *Pm1aSTS1* assay showing specificity to *Pm1a*-containing *Triticum aestivum* lines. CS (Chinese Spring), Avocet R, and Kukri do not have *Pm1a*. CS/Ax7A (Chinese Spring/Axminster*7A), Thew, Norka, and Schomburgk have *Pm1a*.

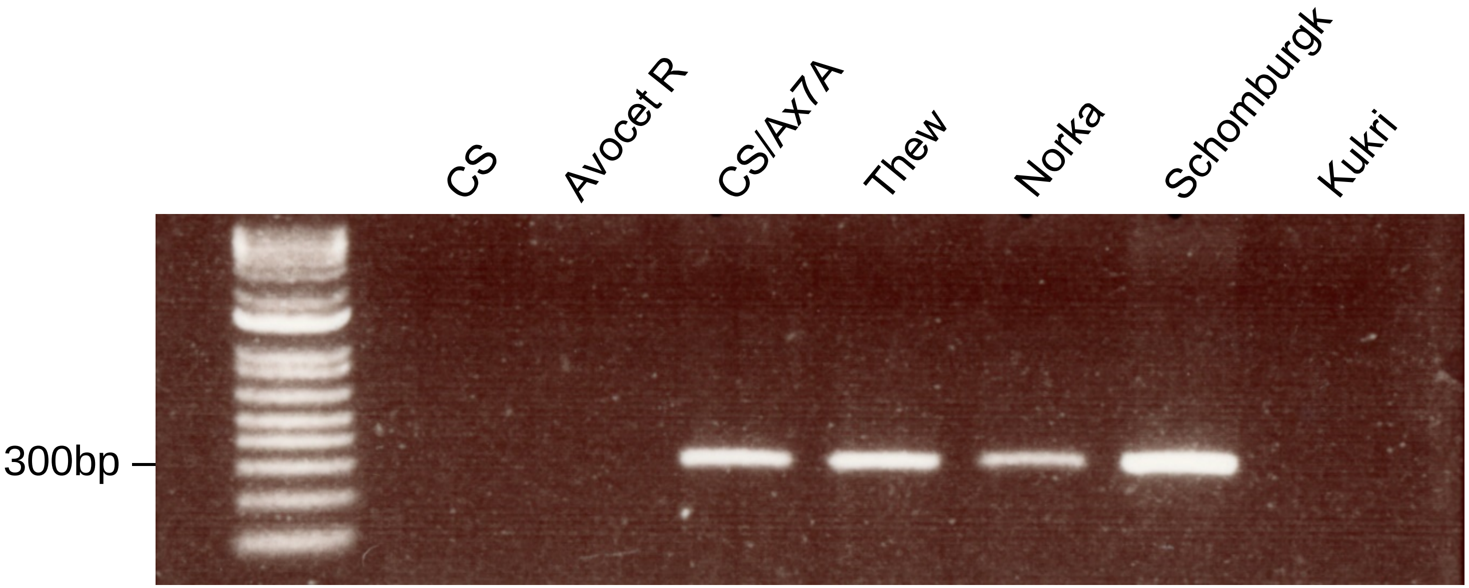

**Fig. S3** Amplification of a large product based on the candidate contig from *Triticum aestivum* cv. Chinese Spring/Axminster*7A (CS/Ax7A) was absent in double mutants of *pm1* and *sr15/lr20*. PCR product used for Sanger sequencing based on the candidate contig #8725 sequence amplified in *Pm1a*-containing lines CS/Ax7A, Thew, Norka and Schomburgk, and point mutant 10487, but not in Thew and CS/Ax7A double mutants (9024-10693) presumed to be deletions.

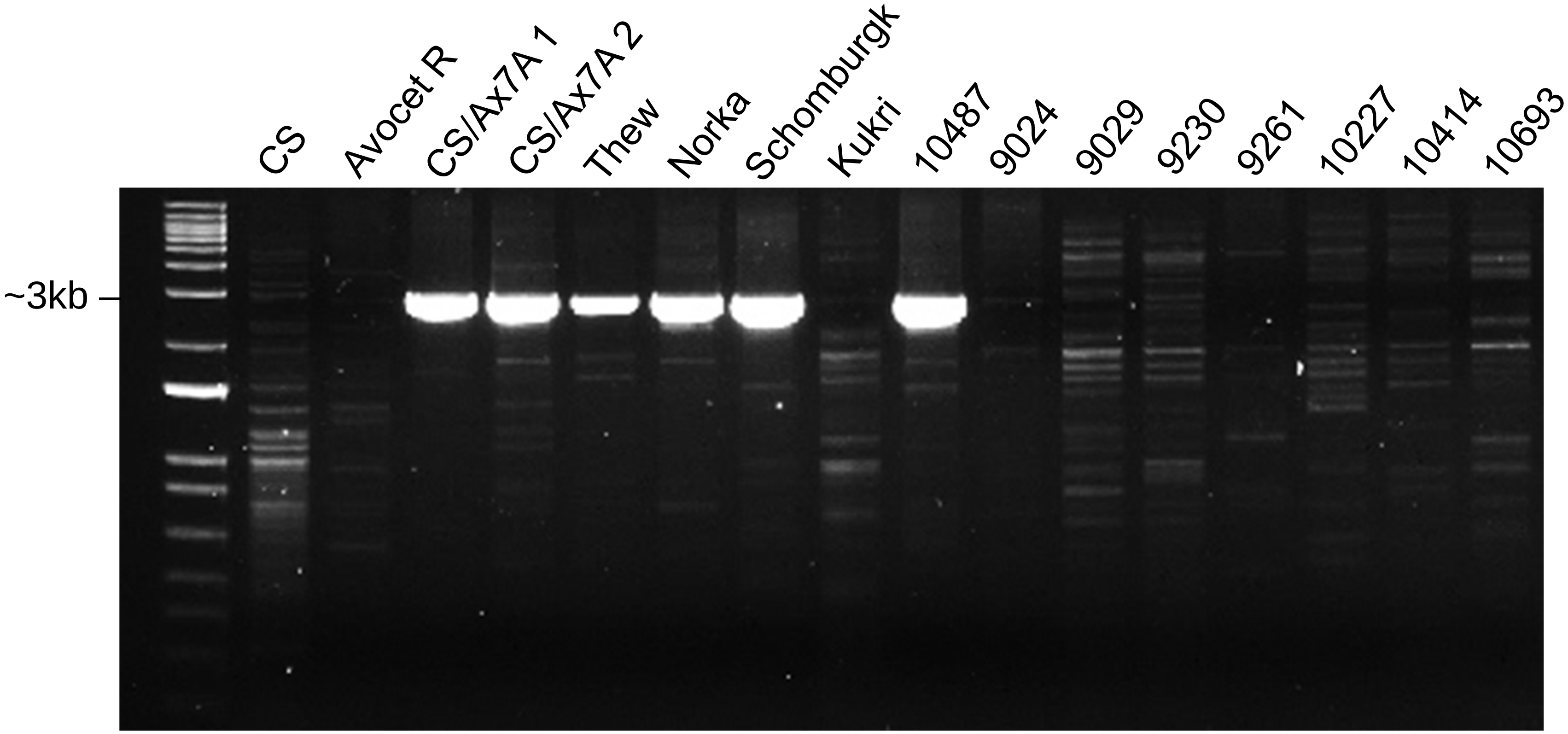

**Fig. S4** Clear differentiation of resistant and susceptible *Triticum aestivum* seedlings in recombinant inbred line populations inoculated with *Blumeria graminis* f. sp. *tritici* (*Bgt*). Three-week-old seedlings at 10 days post-inoculation with the Canberra *Bgt* population. L, susceptible; R, resistant.

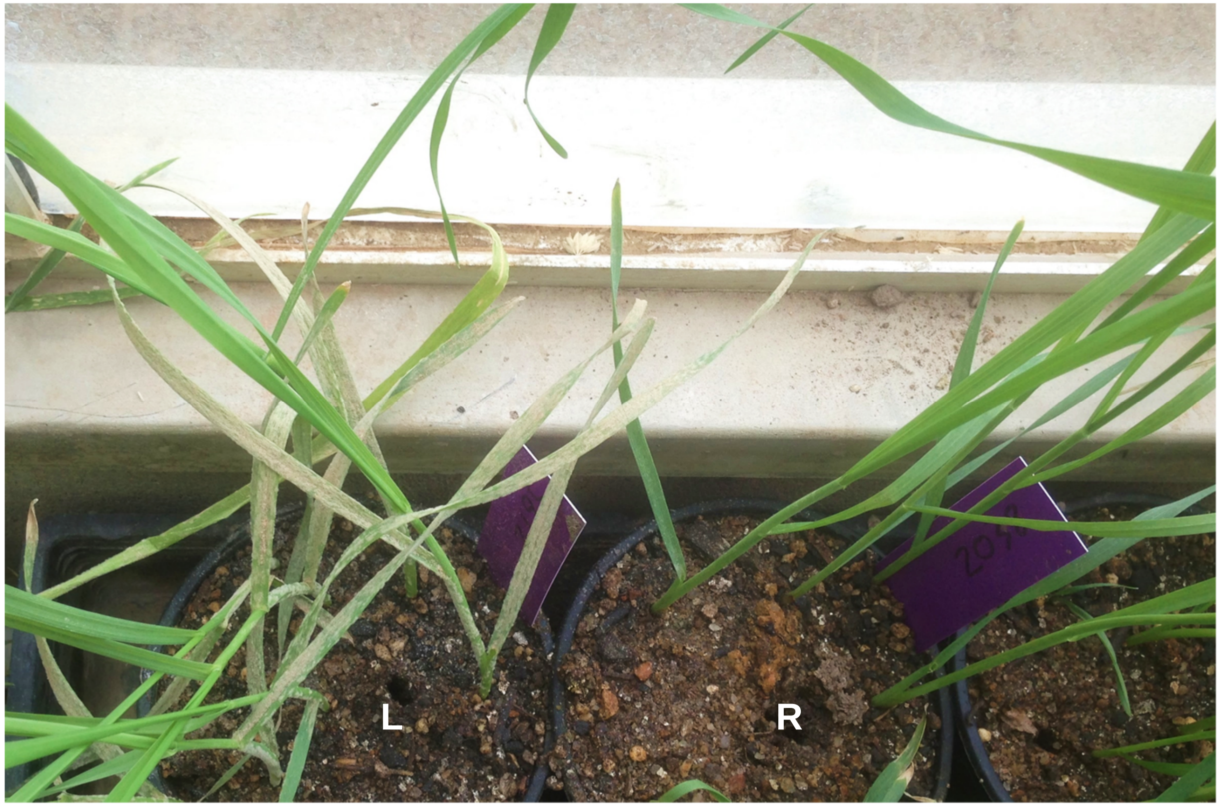

**Fig. S5** Bi-parental mapping population *Blumeria graminis* f. sp. *tritici* (*Bgt*) 96224 (*avrPm1a*) × *B.g. triticale* THUN-12 (*AvrPm1a*) segregates on the *Triticum aestivum* near isogenic line (NIL) Axminster/8*Chancellor containing *Pm1a*. (**a**) Virulence phenotype of the isolates 96224 and THUN-12 on the near-isogenic line (NIL) Axminster/8*Chancellor and the control Chancellor at 10 days post-inoculation from three independent infection experiments. (**b**) Leaf coverage (LC) according to the following scoring: 0 (0-10% LC), 0.25 (10-40% LC), 0.5 (40-60% LC) and 1 (60-10% LC) of the parental isolates 96224 and THUN-12 on Axminster/8*Chancellor on 15 detached leaf segments. The infection score of the 15 detached leaf segments are summarised as boxplots. Individual datapoints are depicted as black dots. Datapoints are jittered for better readability (**c**) Distribution of LC according to the same scoring system as in (b) of 118 $F_1$ progeny of the cross 96224 × THUN-12 on Axminster/8*Chancellor.

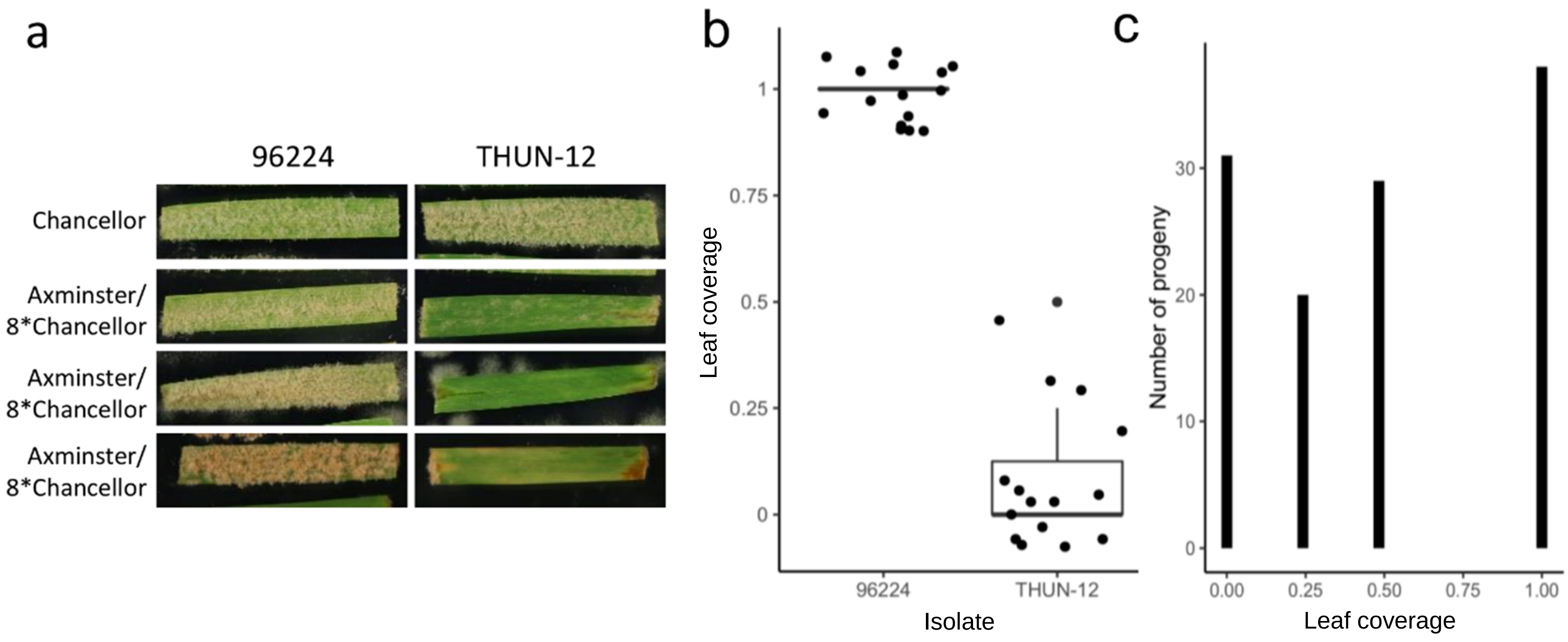

**Fig. S6** *Agrobacterium* mediated transient co-expression of *BgtE-5612* or *BgtE-20015* with *Pm1a* and verification of protein presence in *Nicotiana benthamiana*. (**a**) The effector candidates *BgtE-5612_96224*, *BgtE-5612_THUN12*, *BgtE-20015_96224* and *BgtE-20015_THUN12* were co-infiltrated with *Pm1a* (OD=1.2, ratio 1:4 R:effector) in *N. benthamiana*. Co-infiltration of *Pm3f*$^{L456P/Y458H}$ and *AvrPm3*$^{a2/f2}$ was used as a positive control known to give weak hypersensitive response (HR) (Bourras *et al*. 2019). HR was assessed by fluorescence imaging (Fusion FX imager, see methods) 5 days after infiltration. (**b-c**) Western blot detection of BGTE-20015_THUN12 and BGTE-20015_96224 (b) or PM1a (c) C-terminally fused with a hemagglutinin (HA) epitope tag (upper panel) and Ponceau staining of RuBisCO as a loading control (lower panel). (**d**) C-terminal HA-tagged BgtE-5612 were co-infiltrated with Pm1a (OD=1.2, ratio 1:4 R:effector) in *N. benthamiana*. Co-infiltration of *Pm3f*$^{L456P/Y458H}$ and *AvrPm3*$^{a2/f2}$ was used as a positive control. HR was assessed by fluorescence imaging (Fusion FX imager, see methods) 5 days after infiltration.

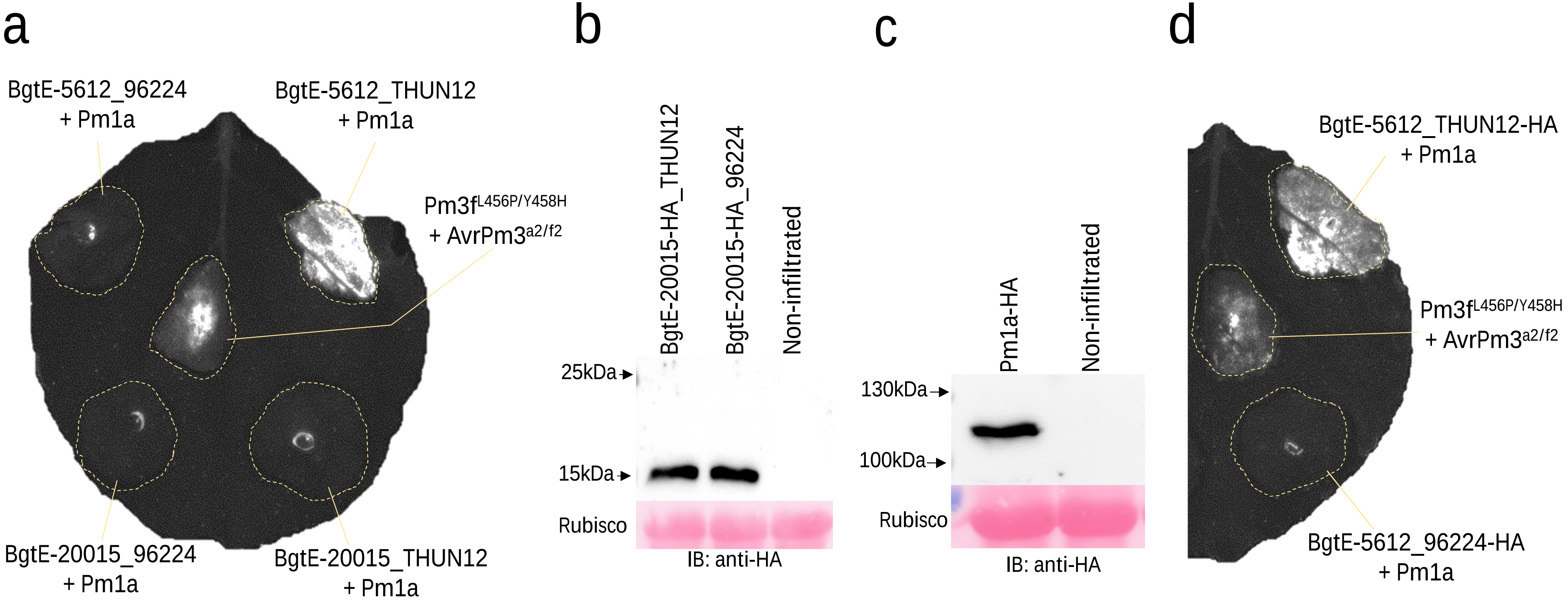

**Fig. S7** Phylogenetic tree of Pm1a with cloned CNL immune receptors from various plant species. The optimal tree with a sum of branch length = 29.96. Distances were computed using the Poisson correction method and are in units of the number of amino acid substitutions per site. Pm1a is indicated by black arrow. General taxonomic relationships are marked by the colour wheel: red, *Pm3* locus group; yellow, barley *Mla* locus group; blue, *Solanaceae* group; orange, *Fabaceae* group; purple, *Oryza sativa* blast group; green, *Arabidopsis* group. Red dots indicate wheat and barley powdery mildew R genes. Navy blue dots indicate *Triticeae* derived genes.

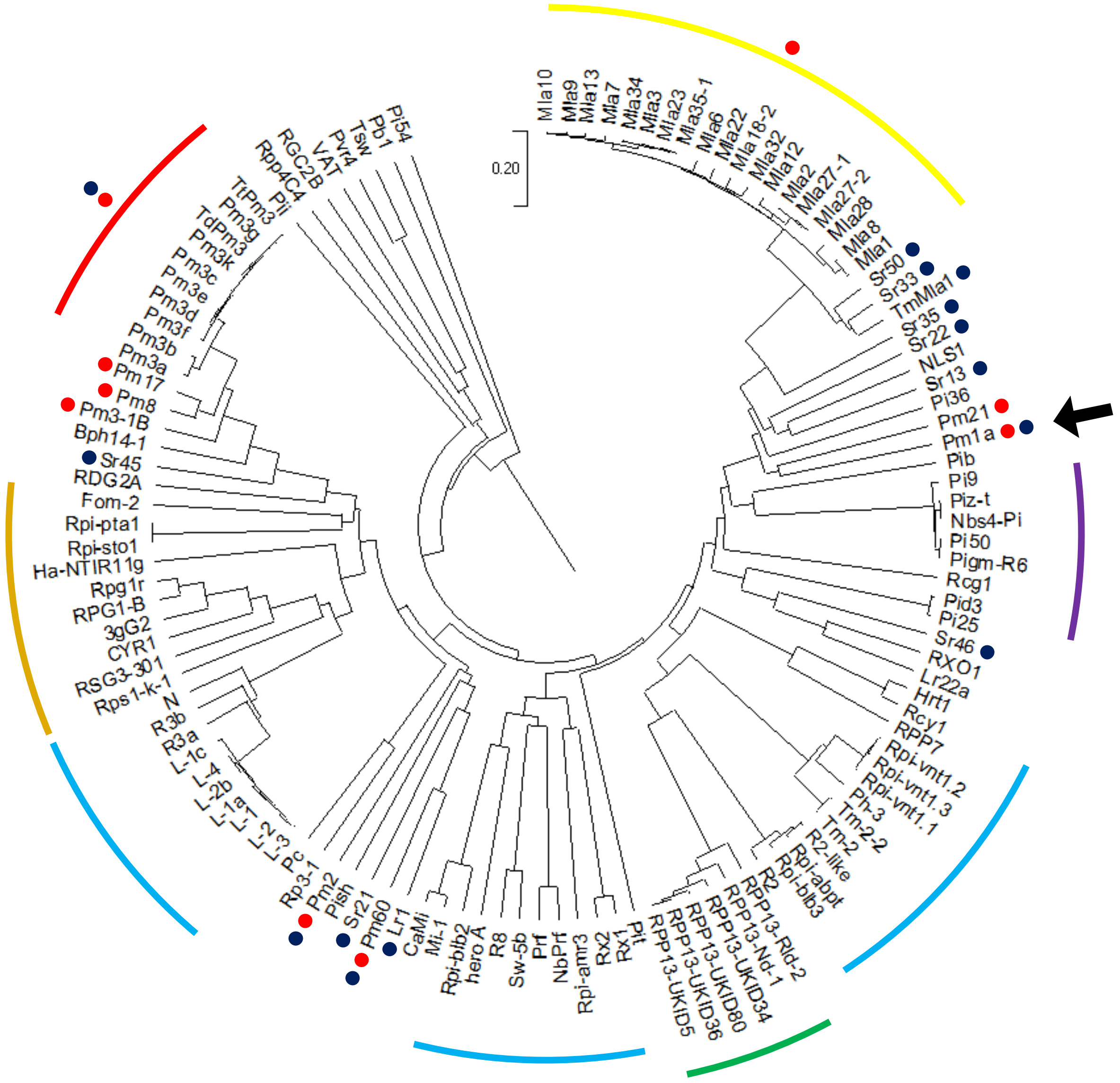

**Fig. S8** Expression of the candidate effector family E004 gene members in the two *Blumeria graminis* isolates *B.g. triticale* THUN-12 and *B.g. tritici* 96224 based on RNAseq data.
Expression levels are depicted as the mean of rpkm (reads per kilo base per million mapped reads) of three biological replicates with standard error bars shown. Expression level is shown at 2 days post-inoculation on susceptible triticale cv. Timbo and *Triticum aestivum* cv. Chinese Spring for THUN-12 (upper panel) and 96224 (lower panel), respectively. RNAseq data were described in previous studies (Praz *et al*., 2018, Menardo *et al*., 2016).

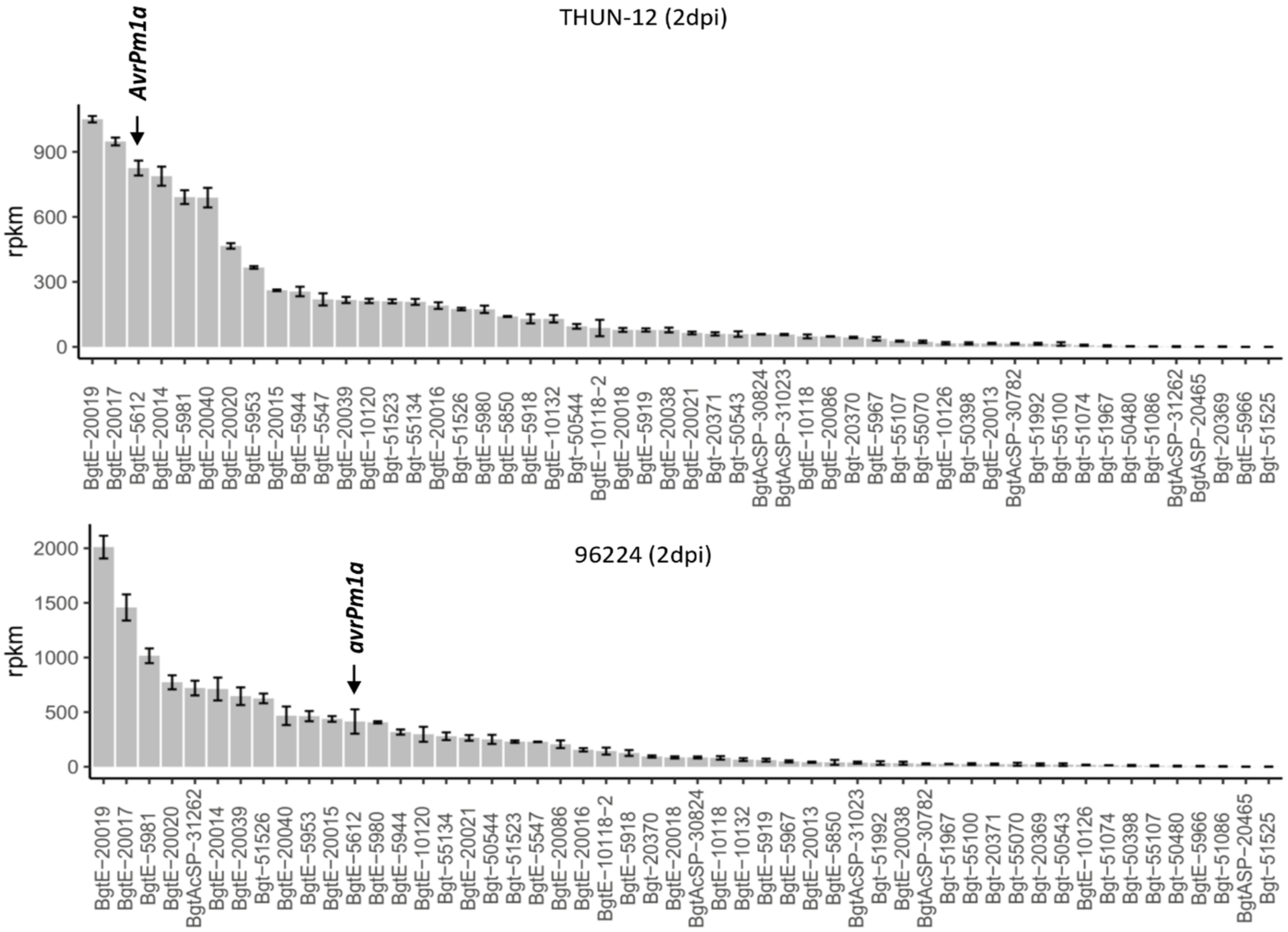

**Fig. S9** Cytological examination of *Triticum aestivum* chromosome 7A in cv. Chinese Spring (CS) and lines carrying *Pm1a*. (**a**) Chromosome 7A FISH karyotypes of CS and four *Pm1a*-carrying lines (CS/Ax7A, Kenya W744, Thew, Norka). Oligo-pSc119.2 and Oligo-pTa535 were labelled with 6-carboxyfluorescein (6-FAM) and 6-carboxytetramethylrhodamine (Tamra), generating green and red signals, respectively, and allowing us to identify individual chromosomes. Chromosomes were counterstained with 4',6-diamidino-2-phenylindole (DAPI) and fluoresced blue. Arrows point to the weak pSc119 green signal at the distal region of 7AL in all four *Pm1a*-carrying lines but was absent in CS. (**b**) Flow-sorted chromosome was confirmed to be 7A of CS/Ax7A based on the above karyotype.

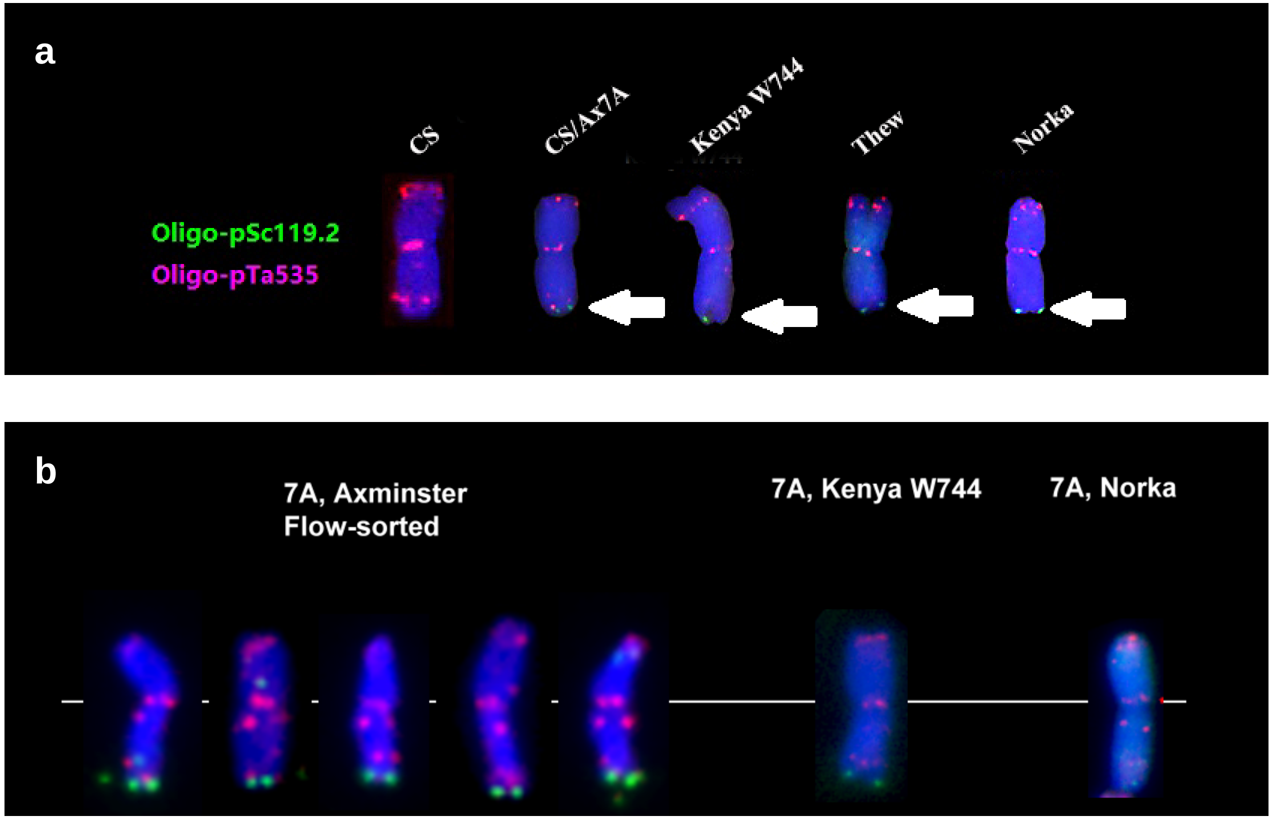

**Fig. S10** *Triticum aestivum* cvs. Chinese Spring (CS) vs. Axminster sequence divergence in distal chromosome 7A.
(**a**) SNP density based on Axminster read alignments in 5 Mb windows along chromosome 7A of CS reference assembly (IWGSC RefSeq v1.0). The region bearing *Pm1* is located at the distal end of 7AL.
(**b**) Alignment scores of IWGSC RefSeq v1.0 annotated genes to Axminster 7A contigs drop sharply at distal end of 7AL. Ordered by position in CS chromosome 7A.

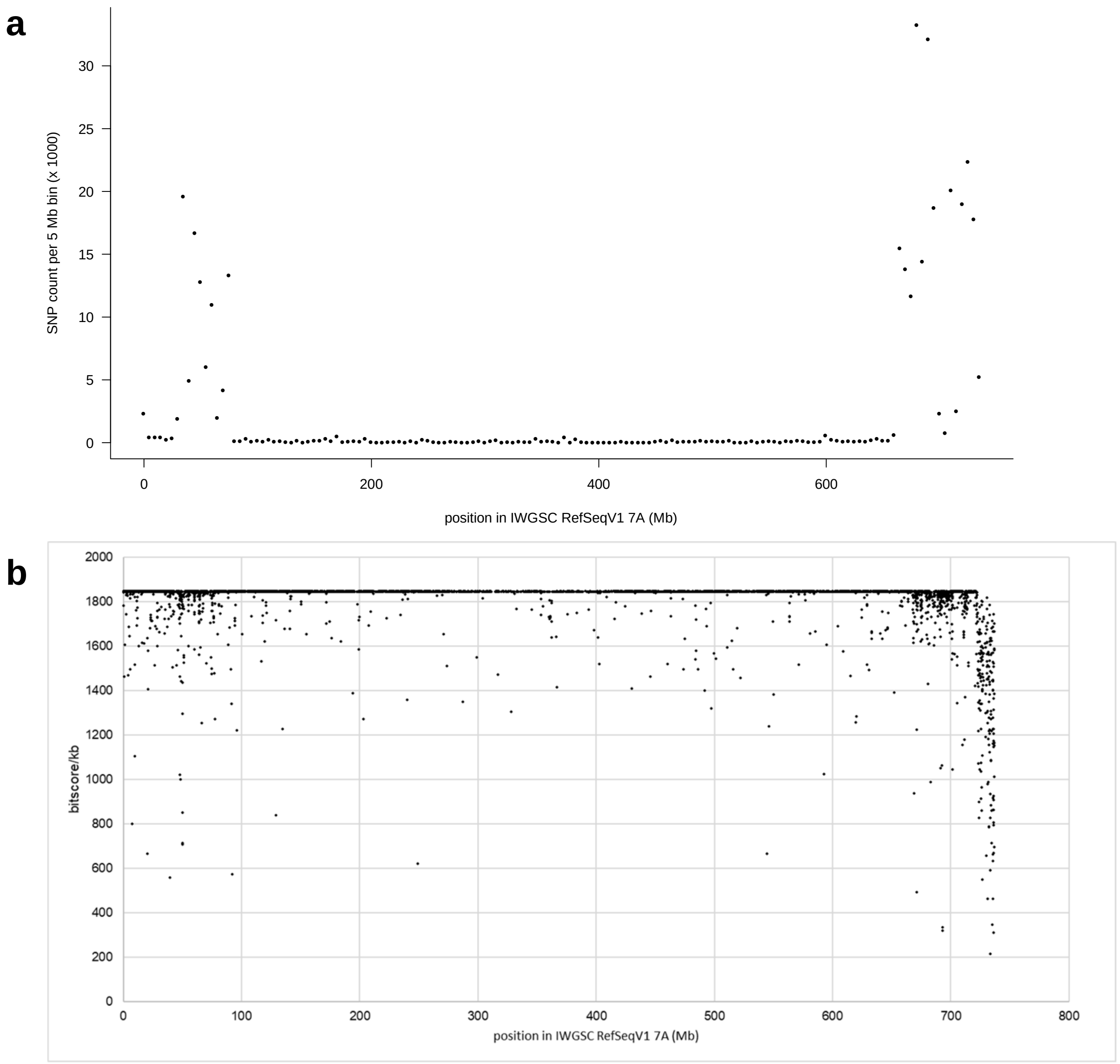

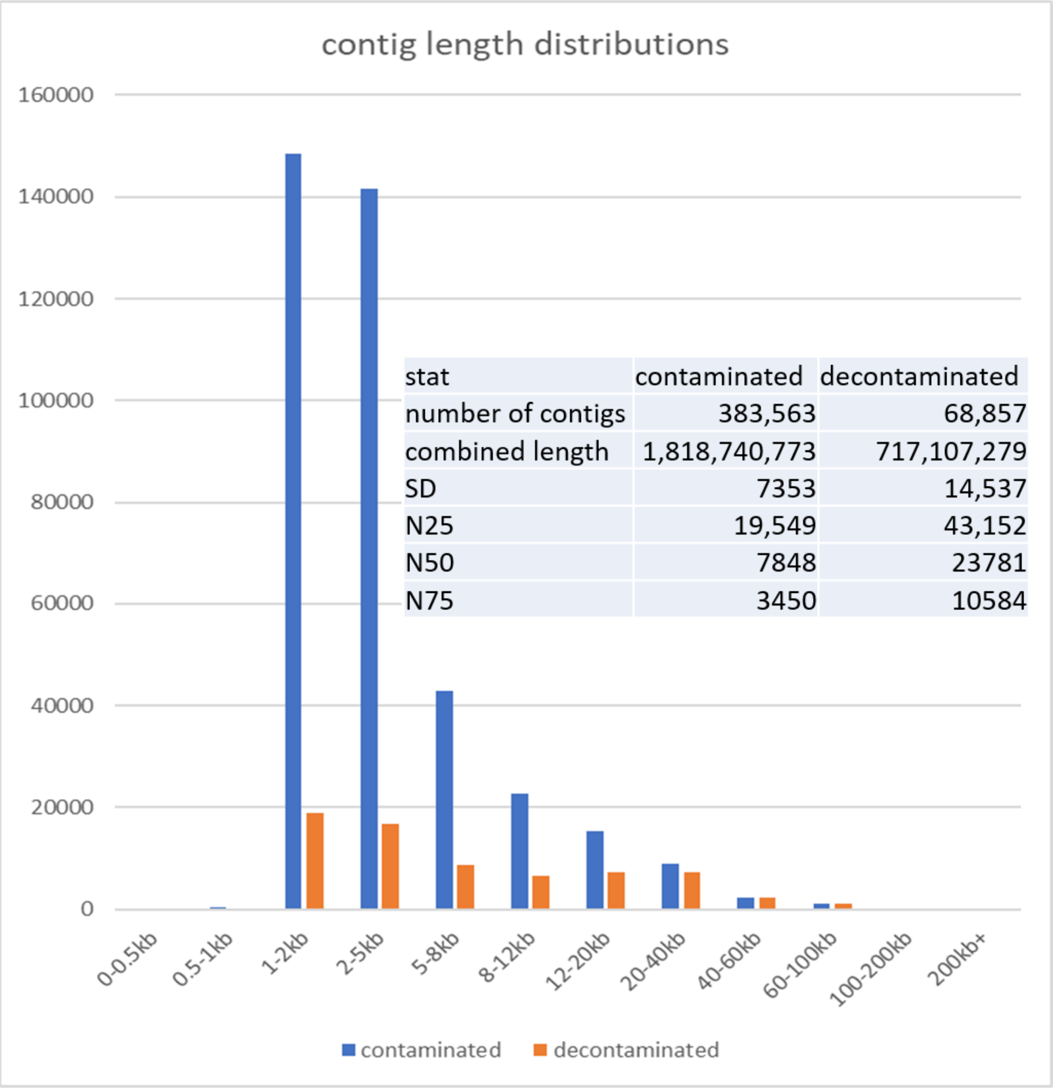


| stat | contaminated | decontaminated |
|---|---|---|
| number of contigs | 383,563 | 68,857 |
| combined length | 1,818,740,773 | 717,107,279 |
| SD | 7353 | 14,537 |
| N25 | 19,549 | 43,152 |
| N50 | 7848 | 23781 |
| N75 | 3450 | 10584 |

**Fig. S11** Contig length distributions from assembly of *Triticum aestivum* cv. Axminster flow-sorted chromosome 7A with and without contaminants.
Number of contigs (y-axis) belonging to each length (kb) bin (x-axis). Blue bars show counts for raw assembly, orange bars show counts for assembly with probable chromosomes 2A and 3A derived contigs removed. Most contamination appeared to be shorter contigs as N50 improved 3-fold with contaminants removed and combined length moved closer to the true size of chromosome 7A (table inset with lengths in bp).

**Fig. S12** Proportions of reads from diploid *Triticeae* species exactly matching to the flow-sorted chromosome 7A assembly of *Triticum aestivum* cv. Axminster. Values are percentage of reads exactly matching (edit distance = 0) out of the total uniquely mapped reads per species. Red dashed lines indicate levels of *T. urartu*, an ancestral benchmark from the A-genome donor. Black arrows indicate levels for *Dasypyrum villosum*, a speculative donor. (**a**) Reads aligned to whole set of Axminster 7A contigs. (**b**) Reads aligned to exclusive set of contigs assigned to distal Axminster 7AL. *D. villosum* levels approach those of *T. urartu*.

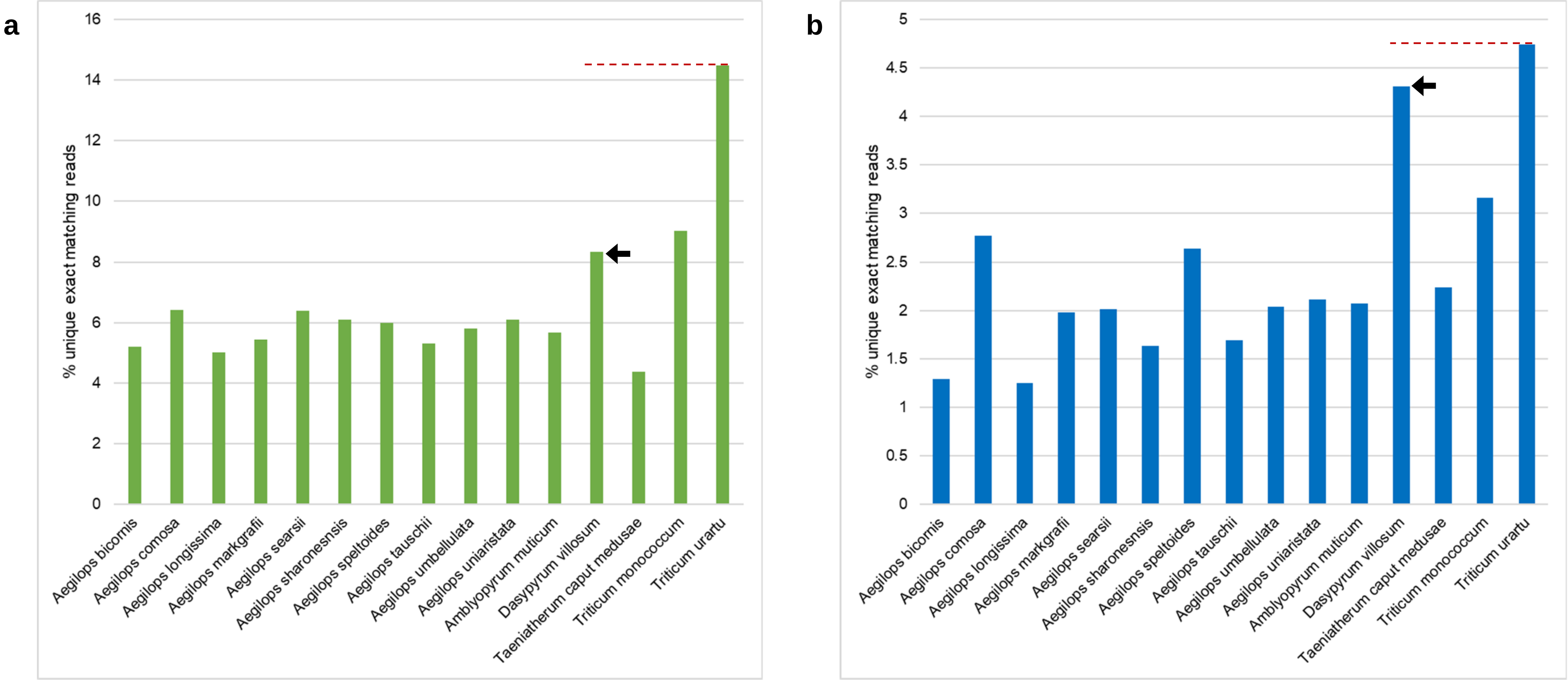

**Fig. S13** Protein alignment of the candidate effector family E004 members in the reference *Blumeria graminis* f. sp. *tritici* isolate 96224. The effector family definition is based on Müller *et al.* (2019). Proteins were aligned using the Clustal algorithm and conserved amino acid residues are coloured according to the ClustalX colour scheme (blue: hydrophobic; red: positively charged; purple: negatively charged; green: polar uncharged; yellow: proline; pink: cysteine; orange: glycine). The Y/FxC motif and conserved C-terminal cysteine are indicated by vertical black boxes. *AvrPm1a* (BGTE-5612) is indicated by a horizontal black box.

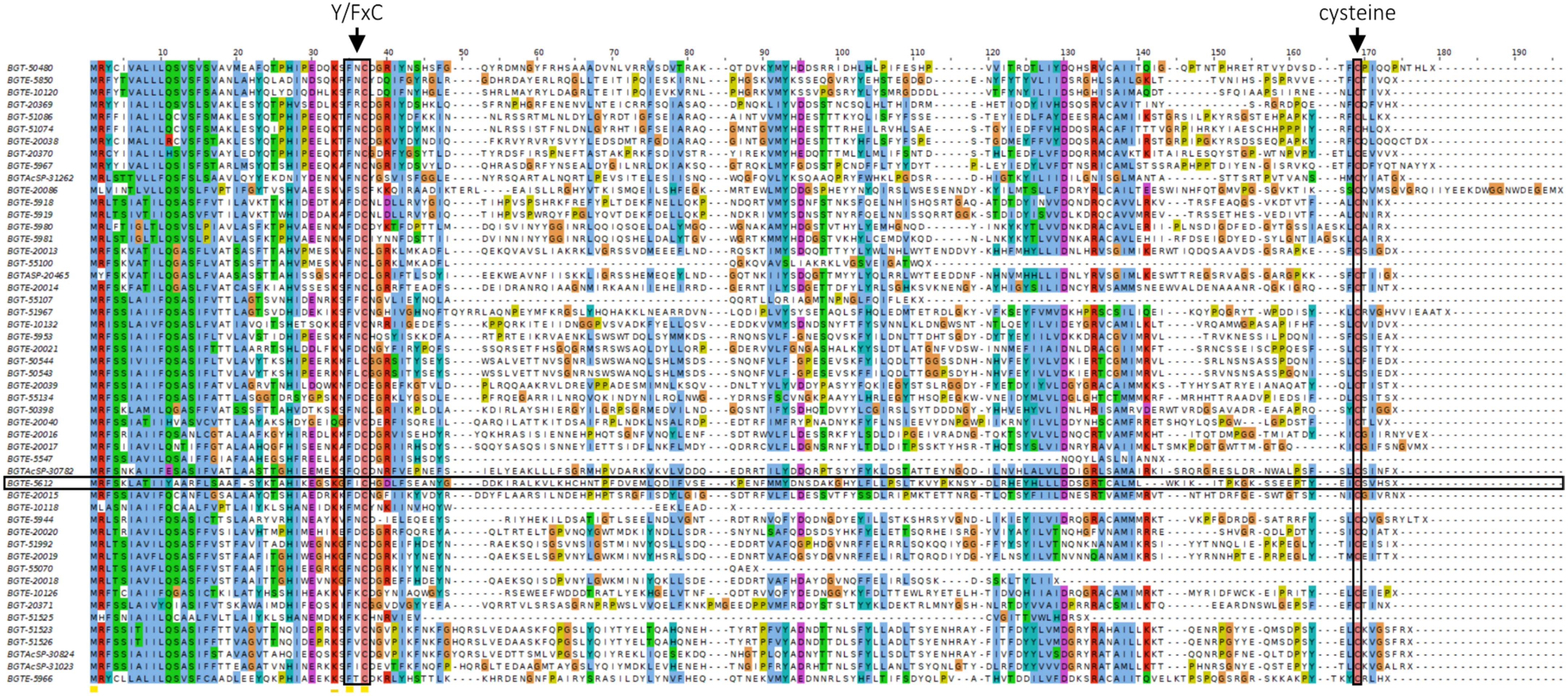

**Fig. S14** (**a**) Alignment of *Blumeria graminis* f. sp. *tritici* AVR proteins and three family members of AVRPM1A. Protein sequences were aligned according to the intron position. Signal peptide, the Y/FxC motif, C-terminal cysteine and intron position are indicated. Intron size in basepairs (bp) is indicated for each protein. Number of amino acid (aa) residues between the intron and the first (part of Y/FxC motif) and the second cysteine are also indicated above the protein sequence. (**b**) Modelling of the 2D protein structure of AVRPM1A_THUN-12 and AVRPM2 without signal peptide using the Quick2D toolkit. The Y/FxC motif, C-terminal cysteine and intron position are indicated. Polymorphic amino acids between AVRPM1A_THUN-12 and AVRPM1A_96224 are highlighted in orange. (**c**) Structural modelling of AVRPM1A_THUN-12 and AVRPM2 using IntFOLD5.0. Domains predicted by the IntFOLD algorithm are depicted as follows: red indicates a predicted β-strand, blue indicates a predicted α-helix. The AVRPM1A protein models to the following two templates; a ribonuclease crystallized from *Aspergillus phoenicis* (PDB id =1rds) (*Nonaka et al*., 1993) and the ribonuclease F1 of *Fusarium moniliforme* (PDB id =1fus) (*Vassylyev et al*., 1993). AVRPM2 models to BEC1054 a RNase-like effector from barley powdery mildew (*B.g. hordei*) and family member of AVRPM2 (PDB id = 6fmb) (*Pennington et al*., 2019) as well as a ribonuclease from *Hericium erinaceus* (PBD id = 5GY6) and the ribonuclease from *Aspergillus phoenicis* (PDB id =1rds).

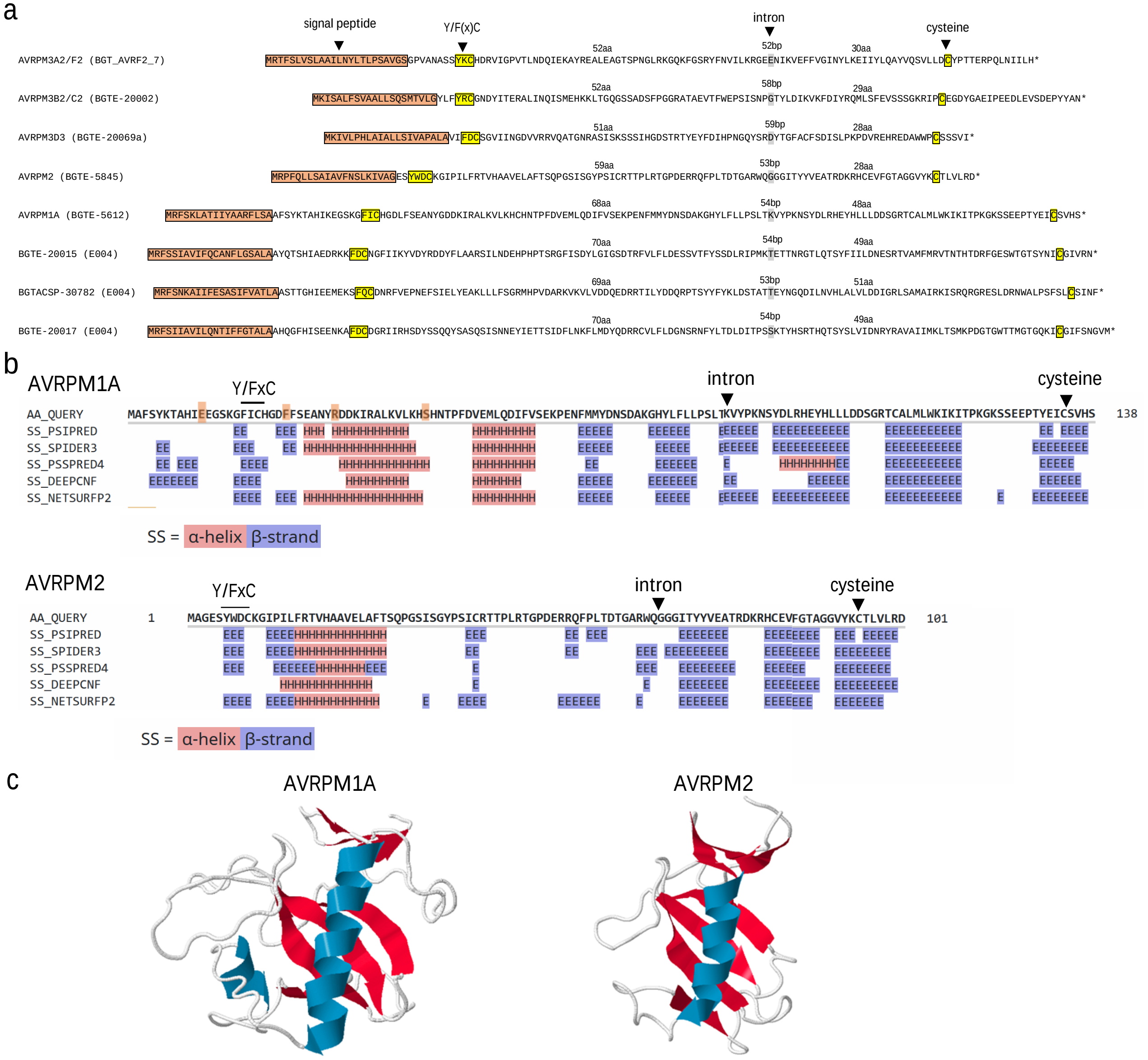

**Table S1** Summary of candidate effector genes and the encoded proteins in the genetic confidence interval underlying the QTL on Bgt_chr-06 in the reference *Blumeria graminis* f. sp. *tritici* isolate 96224.

| Gene Name[1] | SNPs[2] | AA[3] | Effector family[4] | Pfam[5] | Signal peptide[6] | Size[7] | Expression 96224[8] | Expression THUN-12[9] | logFC[10] |
|---|---|---|---|---|---|---|---|---|---|
| BgtE-20040 | - | - | E004 | - | yes (1-21) | 159 | 466.6 | 688.6 | 0.50 |
| BgtE-20016 | - | - | E004 | - | yes (1-21) | 163 | 155.1 | 190.4 | 0.23 |
| BgtE-20017 | - | - | E004 | - | yes (1-21) | 163 | 1457.8 | 947.7 | -0.69 |
| BgtE-5547 | - | - | E004 | - | yes (1-21) | 61 | 22.1 | 20.4 | -0.13 |
| BgtAcSP-30782 | 2 | - | E004 | - | yes (1-21) | 160 | 26.3 | 14.5 | -0.89 |
| BgtE-5612 | 5 | K28E,L40F, G47R,C60S | E004 | - | yes (1-18) | 155 | 414.1 | 825.2 | 0.95 |
| Bgt-20015 | 1 | T94A | E004 | - | yes (1-21) | 160 | 437.6 | 261.2 | -0.81 |

[1]Gene name of effector candidates in *B.g. tritici 96224* reference isolate
[2]Number of SNPs in isolate THUN-12 compared to isolate 96224 based on Illumina resequencing data
[3]Amino acid changes in isolate THUN-12 compared to isolate 96224
[4]Candidate effector family, based on effector family definition in Müller *et al.* (2019)
[5]Pfam domain annotation of mature peptide of effector proteins candidates
[6]Signal peptide prediction based on SignalP3.0, amino acids forming the signal peptide are indicated in brackets
[7]Protein size as number of amino acids
[8]Mean rpkm value of three independent biological replicates of RNAseq data of the isolate *B.g. tritici* 96224 at two days post infection (2dpi)

[9]Mean rpkm value of three independent biological replicates of RNAseq data of the isolate *B.g. triticale* THUN-12 at 2dpi
[10]Log-fold change of expression of isolate THUN-12 in comparison to isolate 96224, logFC-values above 1.5 or below -1.5 are considered significant

**Table S2.** Primers used to clone gene-synthesized *Pm1a* and *Pm1a*-HA into Agrobacterium compatible expression vector pIPKb004

| **Description** | **Forward primer *5'-3'*** | **Forward site** | **Reverse primer *5'-3'*** | **Reverse site** | **Annealing temp. (°C)** | **Polymerase** | **Amplicon length (bp)** |
|---|---|---|---|---|---|---|---|
| amplification of Pm1a-fragmentA[1] | CTATCTCTCTCGAGCTTTCG CAGATC | Vector overlap | ATTAAGCTCACTGGCGTAAAT TCGATG | Intron1 | 65°C | Platinum SuperFi (Invitrogen) | 2637 |
| Amplification of Pm1a-fragmentB[1] | GACCATCGAATTTACGCCAG TGAG | Intron1 | GATCGGGGAAATTCGAGTCAT CAC | Vector overlap | 65°C | Platinum SuperFi (Invitrogen) | 2262 |
| Amplification of Pm1a-fragmentB[1] introducing HA epitope tag | GACCATCGAATTTACGCCAG TGAG | Intron1 | TTAAGCGTAATCTGGAACATC GTATGGGTAGATCACTTGGTC TTCTTGCTGC | Exon3 | 65°C | Platinum SuperFi (Invitrogen) | 2254 |
| Amplification of Pm1a fragmentB-HA reintroducing vector ovelap | GACCATCGAATTTACGCCAG TGAG | Intron1 | GATCGGGGAAATTCGAGTCAT CACCACTTTGTACATTAAGCG TAATCTGGAACATCGTATGG | Exon3-HA | 65°C | Platinum SuperFi (Invitrogen) | 2289 |

[1] template sequence is listed in Supplementary Data File S1

**Table S3** Accession numbers of immune receptor proteins used to construct the phylogenetic tree in Figure S7.

| **Protein accession or DOI** | **R gene** |
|---|---|
| This study | *Pm1a* |
| AAC49408 | *Prf* |
| AAC97933 | *Mi-1* |
| AAF36987 | *Hrt1* |
| AAF42831.1 | *RPP13-Rld-2* |
| AAF42832.1 | *RPP13-Nd-1* |
| AAG31014 | *Sw-5b* |
| AAG37354 | *Mla1* |
| AAO16000 | *Mla13* |
| AAO43441 | *Mla12* |
| AAQ10735 | *Tm-2* |
| AAQ10736 | *Tm-2*$^{2}$ |
| AAQ55540 | *Mla7* |
| AAQ55541 | *Mla10* |
| AAQ96158 | *Pm3b* |
| AAR19096 | *Rpg1-b* |
| AAS49213 | *3gG2* |
| AAS79233 | *Rp3* |
| AAT08955 | *Ha-NTIR11g* |
| AAW48299 | *R3a* |
| AAX31149 | *Rxo1* |
| AAX89382 | *Rps1k-1 and/or Rps1k-2* |
| AAY21626 | *Pm3a* |
| AAY21627 | *Pm3d* |
| AAY33493.1 | *Pi54 (Syn. Pik-k(h))* |
| AAZ23113 | *Pm3f* |
| AAZ95005 | *Rpi-blb2* |
| ABB78077.1 | *Pm3c* |
| ABB78078.1 | *Pm3e* |
| ABB78079.1 | *Pm3g* |
| ABB88855 | *Pi9* |
| ABB91438 | *Fom-2* |
| ABC73398 | *Piz-t* |
| ABC94599 | *Pi2* |
| ABE68835 | *CaMi* |
| ABS29034 | *Lr1* |
| ABY58665.1 | *Pm3k (TdPm3)* |
| ABY58667.1 | *TtPm3* |
| ACB72455 | *Pc* |
| ACI25288.1 | *Rpi-sto1* |

| | |
|---|---|
| ACI25289.1 | *Rpi-pta1* |
| ACJ66594 | *Rpi-vnt1.1* |
| ACJ66595.1 | *Rpi-vnt1.2* |
| ACJ66596 | *Rpi-vnt1.3 (Syn. Rpi-phu1)* |
| ACN56757.1 | *RPP13-UKID80* |
| ACN56765.1 | *RPP13-UKID36* |
| ACN56766.1 | *RPP13-UKID34* |
| ACN56776.1 | *RPP13-UKID5* |
| ACN79513 | *Pid3* |
| ACU65454 | *R2-like* |
| ACU65455 | *Rpi-abpt* |
| ACU65456 | *R2* |
| ACU65457 | *Rpi-blb3* |
| ACZ65484 | *Mla2* |
| ACZ65485 | *Mla3* |
| ACZ65486 | *Mla8* |
| ACZ65487 | *Mla9* |
| ACZ65490 | *Mla18-2* |
| ACZ65492 | *Mla22* |
| ACZ65493 | *Mla23* |
| ACZ65495 | *Mla27-1* |
| ACZ65496 | *Mla27-2* |
| ACZ65497 | *Mla28* |
| ACZ65500 | *Mla32* |
| ACZ65501 | *Mla34* |
| ACZ65502 | *Mla35-1* |
| ADB07392 | *Bph14* |
| ADF29624 | *Pi36* |
| ADK47521 | *Rdg2a* |
| ADU57957 | *CYR1* |
| ADX06722 | *TmMla1* |
| AEC47890 | *R3b* |
| AER13157 | *Rpp4C4* |
| AFM35701 | *Pi25* |
| AGI99538 | *RSG3-301* |
| AGQ17386 | *Sr33* |
| AGP75918 | *Sr35* |
| AGT37271 | *RPP7* |
| AGY30894 | *Pm8* |
| AGY30895 | *Pm3-1B* |
| AIB02970 | *Ph-3* |
| AIC32313 | *Rpg1r* |

| | |
|---|---|
| AIU36098 | *VAT* |
| AKS24975.1 | *Pi50* |
| ALO61074 | *Sr50* |
| AMY98955 | *Rpi-amr3i* |
| ANJ02805 | *R8 (Syn. Rpi-smira2)* |
| ANZ78204 | *Pvr4* |
| AOR08328 | *Tsw* |
| APF29096 | *PigmR (Syn. PigmR6)* |
| ARO38245.1 | *Lr22a* |
| ATE88995 | *Sr13* |
| AUO29720 | *Pm60* |
| AVK42833 | *Sr21* |
| AVR54589 | *Pm21* |
| AYD60116 | *Pm17* |
| AYV61514 | *Sr46* |
| BAA76282 | *Pib* |
| BAC67706 | *Rcy1* |
| BAH20862 | *Pit* |
| BAJ25849 | *Pb1* |
| BAJ33559 | $L^{3}$ |
| BAJ33561 | $L^{1}$ |
| BAJ33562 | $L^{1a}$ |
| BAJ33563 | $L^{1c}$ |
| BAJ33564 | $L^{2}$ |
| BAJ33565 | $L^{2b}$ |
| BAJ33566 | $L^{4}$ |
| BAM17521 | *N'* |
| BAN59294 | *Pii* |
| CAB50786 | *Rx1* |
| CAB56299 | *Rx2* |
| CAC29241 | *Mla6* |
| CAL64731 | *Rcg1* |
| CUM44200.1 | *Sr22* |
| CUM44213.1 | *Sr45* |
| CZT14023.1 | *Pm2* |
| NP_001067618 | *NLS1* |
| NP_001172592 | *Pish* |
| NP_001233995 | *Hero A* |
| Q9ZSD1 | *RGC2B (Syn. Dm3)* |
| https://doi.org/10.1046/j.1365-313X.2002.029005569.x | *NbPrf (Niben101Scf00650g02002XLOC)* |

**Table S4** Primers used for PCR and sequencing of *Pm1a*.

| **Description** | **Forward primer *5'-3'*** | **Forward site** | **Reverse primer *5'-3'*** | **Reverse site** | **Annealing temp. (°C)** | **Polymerase** | **Amplicon length (bp)** |
|---|---|---|---|---|---|---|---|
| STS dominant marker *Pm1aSTS1* | CAATATAAACTTCAGATGTTCT ATTCTCAAAC | Intron1 | CTACATTGGCTATGCGTGTAG TC | Exon2 | 55 | GoTaq® | 333 |
| EST marker *Pm1aEST1* | TAGCCAATAAACCTCCAAGCGC AGC | Exon2 | GAAGCTCCGTGATCCGTAGTC CATG | Exon2 | 59 | GoTaq® | 732 |
| EST marker *Pm1aEST2* | CGGCTCTGGTCTCAAGAATACT G | Exon3 | GTTCAGTTCAGTTTGTCGCCC | Exon3 | 55 | GoTaq® | 331 |
| Large amplicon covering contig #8725 | ATACTGTTGTATGACTTTATAAG GTCTTGTGC | Intron1 | GTTCAGTTCAGTTTGTCGCCC | Exon3 | 66 | Phusion® | 2964 |
| Primary bridging of contigs #3966 and #8725 | GGGCTGGGAAAGACCAC | Exon1 | CCAGTTGAGAAGGGCATAAAC | Exon2 | 62 | Phusion® | 2282 |
| Secondary bridging of contigs #3966 and #8725 (nested PCR on 1µL 1:50 dilution of primary product) | ACCACCCTCGCCATGG | Exon1 | CATGCAGTCACTTTATTAATTA TTCG | Intron1 | 61 | Phusion® | 1664 |
| Primary amplification of Exon1 | CTCCGTCATCGGCAAGC | Exon1 | CTAGCCAAGAGCAGGTTTTCA CAT | Intron1 | 63 | Phusion® | 1556 |
| Secondary amplification of Exon1 (nested PCR on 1µL 1:50 dilution of primary product) | GACATGGAGGAGTGCATCGAC | Exon1 | CCGCAAGCCCACCACCAAC | Intron1 | 66 | Phusion® | 1006 |
| Sequencing primer 1 | CTCCGTCATCGGCAAGC | Exon1 | n/a | n/a | n/a | n/a | n/a |
| Sequencing primer 2 | GCATCGACCGCTTCATG | Exon1 | n/a | n/a | n/a | n/a | n/a |
| Sequencing primer 3 | n/a | n/a | TGTCTTCTTCACGAACCTGGG CC | Exon1 | n/a | n/a | n/a |
| Sequencing primer 4 | n/a | n/a | CGGGCCTTGATTGATTTACAA TATAATC | Exon1 | n/a | n/a | n/a |
| Sequencing primer 5 | GAGACTATCAGTGTCGAGCTTC C | Exon1 | n/a | n/a | n/a | n/a | n/a |

| | | | | | | | |
|---|---|---|---|---|---|---|---|
| Sequencing primer 6 | n/a | n/a | AAAGCCACCATCTAGGCAAT | Intron1 | n/a | n/a | n/a |
| Sequencing primer 7 | CAATATAAACTTCAGATGTTCT ATTCTCAAAC | Intron1 | n/a | n/a | n/a | n/a | n/a |
| Sequencing primer 8 | n/a | Exon2 | GTTCAGTTCAGTTTGTCGCCC | n/a | n/a | n/a | n/a |
| Sequencing primer 9 | GTTTATGCCCTTCTCAACTGG | Exon2 | n/a | n/a | n/a | n/a | n/a |
| Sequencing primer 10 | GGATCTCCCACACCACTTGAAG ACC | Exon2 | n/a | n/a | n/a | n/a | n/a |
| Sequencing primer 11 | n/a | n/a | GAAGCTCCGTGATCCGTAGTC CATG | Exon2 | n/a | n/a | n/a |
| Sequencing primer 12 | CTCATGGATGCAGCCTGGGGAT TTG | Exon2 | n/a | n/a | n/a | n/a | n/a |
| Sequencing primer 13 | n/a | n/a | CGTCGGAGTGCTGGCATAGC | Exon2 | n/a | n/a | n/a |
| Sequencing primer 14 | GCTATGCCAGCACTCCGACG | Exon2 | n/a | n/a | n/a | n/a | n/a |
| Sequencing primer 15 | n/a | n/a | GATTTAGAAAATAGCTTAGGT GAGCAAG | Exon3 | n/a | n/a | n/a |
| Sequencing primer 16 | n/a | n/a | ATCCGTACTCTTCAACTAACTT ACAG | Exon3 | n/a | n/a | n/a |
